# Near-infrared light triggered in situ release of CO for enhanced therapy of glioblastoma

**DOI:** 10.1186/s12951-023-01802-9

**Published:** 2023-02-09

**Authors:** Juan Ge, Miaomiao Zuo, Qirong Wang, Zhen Li

**Affiliations:** College of Health Science and Engineering, College of Chemistry and Chemical Engineering, Hubei University, Wuhan, 430062 China

**Keywords:** Carbon monoxide, Photodynamic therapy, Anti-inflammatory, Upconversion nanoparticles, Glioblastoma

## Abstract

**Background:**

Photodynamic therapy (PDT) features high biocompatibility and high spatiotemporal selectivity, showing a great potential in glioblastoma (GBM) treatment. However, its application was restricted by the poor therapeutic efficacy and side effect.

**Results:**

In this study, a therapeutic nanoplatform (UCNPs@Ce6/3HBQ@CM) with combination of PDT and CO therapy was constructed, in which a photoCORM and a photosensitizer were loaded onto the surface of upconversion nanoparticles (UCNPs) functioning as photon transducer. Benefitting from NIR excitation and multicolor emission of UCNPs, the penetration depth of excitation light is enhanced and meanwhile simultaneous generation of CO and ROS in tumor site can be achieved. The as-prepared nanocomposite possessed an elevated therapeutic efficiency with the assistance of CO through influencing mitochondrial respiration and depleting ATP, accompanying with the reduced inflammatory responses. By wrapping a homologous cell membrane, the nanocomposite can target GBM and accumulate in the tumor site, affording a powerful tool for precise and efficient treatment of GBM.

**Conclusion:**

This therapeutic nanoplatform UCNPs@Ce6/3HBQ@CM, which combines PDT and CO therapy enables precise and efficient treatment of refractory glioblastoma.

**Supplementary Information:**

The online version contains supplementary material available at 10.1186/s12951-023-01802-9.

## Introduction

Glioblastoma (GBM), a grade IV glioma, is the most aggressive form of malignant gliomas among adults with a low survival rate, poor prognosis, and high risk of recurrence [1]. The current standard treatment involved in GBM patients is surgery followed by radiotherapy plus concomitant and adjuvant chemotherapy [2]. However, the prognosis of GBM remains poor with a median survival of 12–15 months after the final diagnosis and a 5-year life expectancy of less than 10% [3, 4]. Thus, to prolong the overall survival of patients with glioma, there is an urgent need to design new therapeutic modalities for efficient GBM therapy. Photodynamic therapy (PDT) has been developed as a promising anti-tumor technique due to its high selectivity, low toxicity and negligible drug resistance, in which photosensitizers (PSs) are activated by laser irradiation with specific wavelength to produce cytotoxic reactive oxygen species (ROS), leading to cell damage and even apoptosis [5]. As a typical non-invasive light-excited treatment modality, PDT has been approved for clinical use and successfully applied for various tumor such as skin cancer, lung cancer and esophageal cancer [6, 7]. Nonetheless, the poor therapeutic effects of the single mode originated from the diversity, heterogeneity and recurrence of tumors restrict the application of PDT for GBM treatment. In addition, some low active oxygen species generated during PDT, such as H_2_O_2_, is inflammatory factors, whose long-term existence may induce tumor recurrence and metastasis as well as the inflammation of its surrounding tissue [8]. Finding a feasible method to address these issues is of great importance for improving PDT efficiency of GBM treatment [9].

Carbon monoxide (CO), as an endogenous gas molecule, plays a key role in various physiological processes including antithrombotic, antibacterial and anti-inflammatory [10–12]. In recent years, CO has been applied for tumor treatment in an O_2_-free manner under hypoxia condition, which is a major factor limiting the efficiency of PDT [13, 14]. CO has also been confirmed as an effective agent for anti-inflammation [15], thereby decreasing the PDT side effects. Thus, we infer that combination of CO therapy and PDT may be an effective approach for treating GBM. Because of the strong affinity of CO toward hemoglobin to cause severe systemic toxicity, developing ideal therapeutic platform with accurate and controllable delivery of CO to tumor sites is a challenging. Currently, methods for CO generation in tumor site are mainly divided into two types: photo-catalytic reduction of CO_2_ to CO and liberation from stimuli-responsive CO releasing molecules (CORMs). Photo-catalytic reduction system is normally complex and there is a lack of efficient catalysts [16, 17]. Various stimuli-responsive CORMs have been developed, which can be triggered by endogenous stimuli or external factors such as light irradiation. Endogenous stimuli responsive CORMs always suffer from uncontrollable release of CO [18–20]. In contrast, light-triggered CO release from a photo-activated CORM (photoCORMs) has demonstrated appealing profile, which facilitates a precise spatial and temporal control over the CO liberation [21–23]. A few molecular photoCORMs have already been synthesized and studied on their CO-releasing behaviors [24–28], several of which were used for cancer gas therapy [27, 28]. However, since these molecules are activatable only by visible light, they are not competent for GBM treatment due to limited light penetration.

**Scheme 1:**
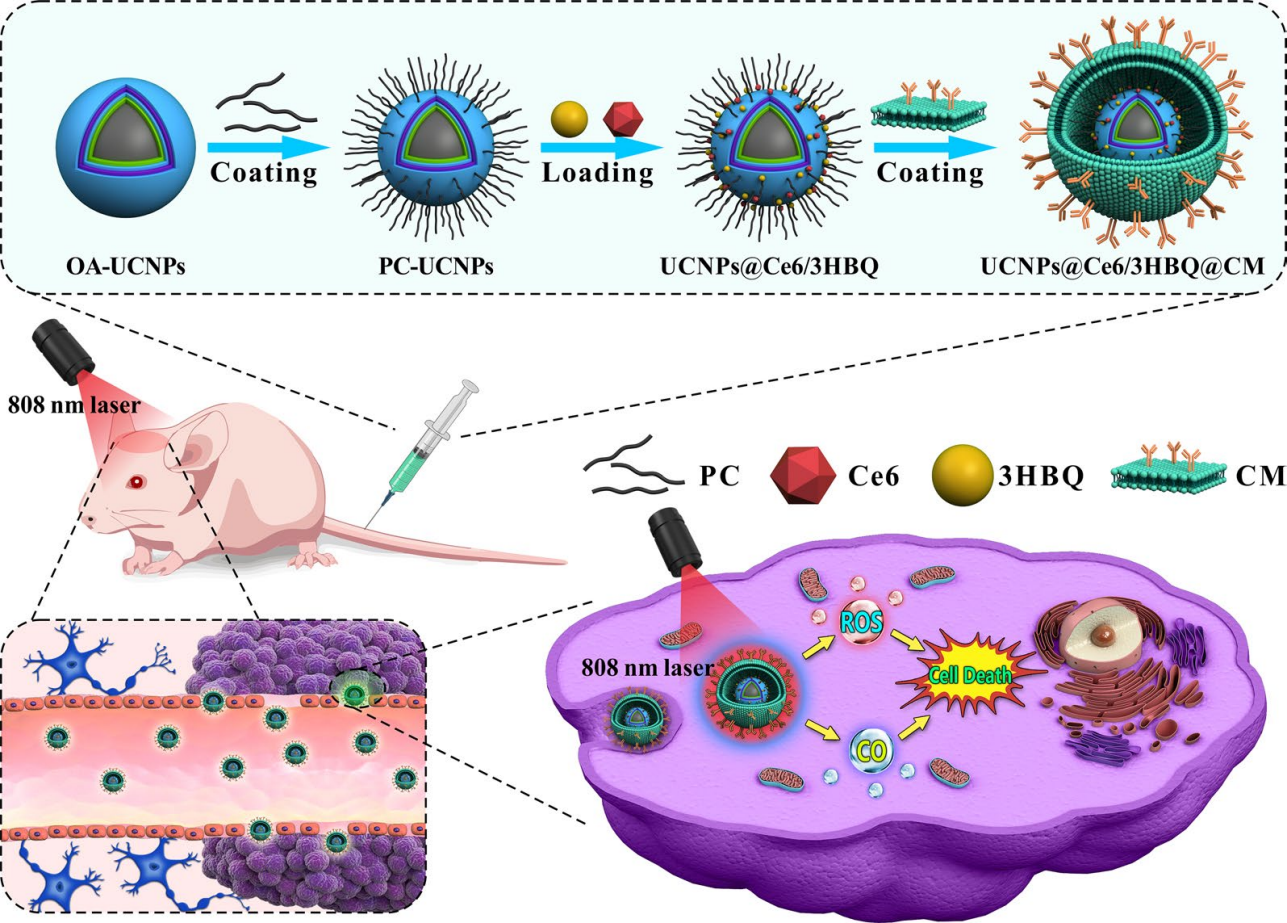
Schematic illustration of the synthesis process of lanthanide-doped nanoparticles loaded with photosesitizer Ce6 (chlorin e6) and photoCORM 3HBQ encapsulated by brain tumor cell membranes (UCNPs@Ce6/3HBQ@CM) and the anti-glioma mechanism (OA and PC represent oleic acid and phosphatidylcholine, respectively)

Herein, we propose a therapeutic nanoplatform for simultaneously activating CO and ROS releasing by an upconverted light transducer, in which a photoCORM (3HBQ) and a photosensitizer (Ce6) are loaded onto the surface of upconversion nanoparticles (UCNPs) through hydrophobic interaction (Scheme 1). In this system, lanthanide doped UCNPs serve as the upconverted light transducers to absorb 808-nm NIR photons and convert them into blue and red photons, which trigger 3HBQ to release CO and activate Ce6 to produce ROS, respectively, thus realizing combined PDT and gas therapy. Taking the advantages of NIR excitation and multicolor emission of UCNPs, the penetration depth of excitation light is enhanced and meanwhile simultaneous generation of CO and ROS in tumor site can be achieved. Besides, efficient upconversion luminescence under NIR excitation can be utilized to track the therapeutic platform in vivo [29–35]. As far as we know, this is the first GBM therapy platform in which both PDT and CO release is simultaneously triggered by NIR light. UCNPs also possess excellent photo/chemical stability and biocompatibility, benefitting the phototherapies and in vivo observations [36]. By constructing such a platform, we anticipate a significantly improved efficacy of GBM treatment compared with single PDT mode by the assistance of CO through interfering with mitochondrial respiration and inducing ATP depletion accompanying with the reduced inflammatory reactions [37–39]. Furthermore, via encapsulating the nanocomposite with homotypic cancer cell membranes, the therapeutic agents are expected to target GBM and enrich in the tumor tissue through homologous targeting [40, 41]. As such, the obtained therapeutic nanoplatform may hold potential in biomedical application and further clinical translation.

## Results and discussion

### CO-releasing performance of 3HBQ

First, we synthesized a photo-activated CO-releasing molecule 3HBQ according to the synthetic routes reported in literature [42], which was characterized with ^1^H-NMR, ^13^C-NMR and HRMS (Additional file 1: Fig. S1–S3). As shown in Additional file 1: Fig. S4a, 3HBQ possessed a strong and broad absorption with two peaks around 450 nm and 478 nm, which matched well with the blue emission of Tm^3+^ (the emitting ion of UNCPs, vide infra) originating from the transition of ^1^D_2_ → ^3^F_4_ and ^1^G_4_ → ^3^H_6_. Under irradiation with a 450-nm LED light source, UV–vis absorption of 3HBQ quickly decreased with prolonging irradiation time due to the change of the conjugation structure after releasing CO (Additional file 1: Fig. S4a, b). Meanwhile, the color of the solution changed from pale yellow to colorless within 10 min, revealing the fast release of CO (Additional file 1: Fig. S4c). In addition, 3HBQ possesses fluorescence emission around 523 nm and 600 nm, which was also weakened after irradiation (Additional file 1: Fig. S5). To gain more direct evidence of the release of CO from 3HBQ, a reported CO probe, FL-CO that can respond to CO in the presence of PdCl_2_ and emit a green fluorescence emission with 500-nm light excitation[43], was utilized to detect the released CO in solution (Additional file 1: Fig. S6). As shown in Additional file 1: Fig. S7, the fluorescence intensity of FL-CO was enhanced upon the irradiation. These results jointly demonstrated that 3HBQ can indeed be activated with photon and release CO. Moreover, by analyzing the structure of the product with HRMS (Additional file 1: Fig. S8), the mechanism of CO release from 3HBQ was illustrated in Additional file 1: Fig. S9.

### Fabrication of the photon transducer

**Fig. 1:**
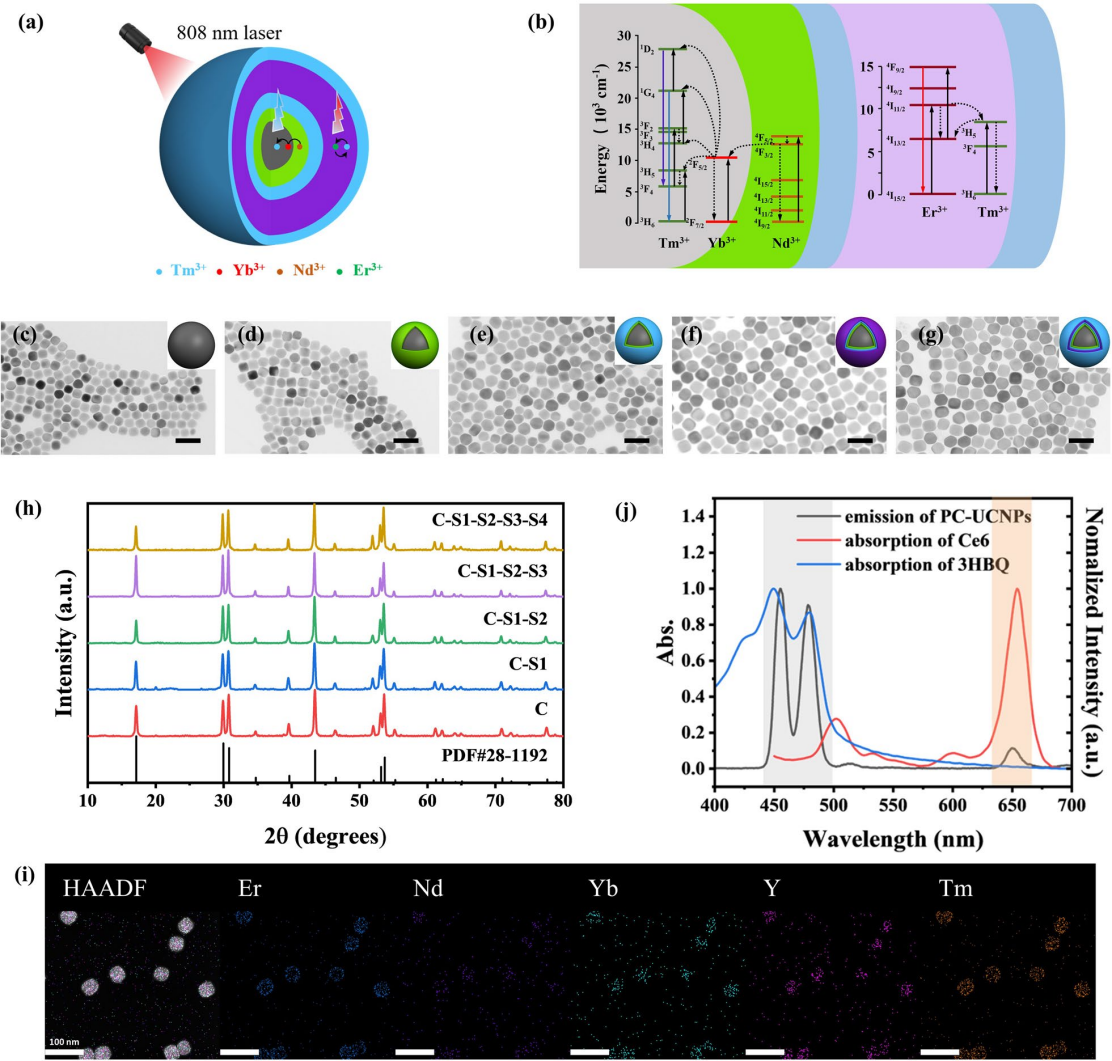
Fabrication and characterization of photon transducer. The structure **a** and energy transfer process **b** of UCNPs. TEM images **c**–**g** and XRD patterns **h** of C, C-S1, C-S1-S2, C-S1-S2-S3 and C-S1-S2-S3-S4 structured UCNPs. Scale bar: 100 nm. **i** HAADF image and corresponding elemental mapping of the obtained UCNPs with C-S1-S2-S3-S4 structure. Scale bar: 100 nm. **j** Absorption spectra of 3HBQ and Ce6, and emission spectrum of PC-UCNPs under excitation at 808 nm

We prepared oleic acid (OA)-coated UCNPs by a seed-mediated method with a C-S1-S2-S3-S4 (C = core, S = shell) structure, i.e. NaYF_4_:Yb/Tm@NaYF_4_:Nd@NaYF_4_@NaErF_4_:Tm@ NaYF_4_ (Fig. 1a). Tm^3+^ was doped in the core (NaYF_4_:Yb/Tm) to obtain strong blue emission around 450 nm and 478 nm, ensuring efficient light-induced generation of CO. The S1 layer doped with Nd^3+^ was designed to harvest the excitation energy of 808-nm photons and transfer it to the core (Fig. 1b). In order to gain red emission around 650 nm under 808-nm laser excitation to activate the PDT, NaErF_4_ was utilized as the matrix in S3 and Tm^3+^ was doped to promote the red emission of Er^3+^ through energy trapping [44]. Two inert NaYF_4_ layers were deposited as S2 and S4, which were able to alleviate the deleterious cross-relaxation between Nd^3+^ and Er^3+^ and protect the emission of UCNPs from quenching caused by the solvent, respectively. Transmission electron microscopy (TEM) images of the as-prepared nanoparticles showed their size evolution: from the core with an average diameter of ~ 32.2 nm to the C-S1 structure (~ 35.0 nm), C-S1-S2 structure (~ 42.8 nm), C-S1-S2-S3 structure (~ 50.7 nm) and finally to the C-S1-S2-S3-S4 structure (~ 56.7 nm) (Fig. 1c–g), Additional file 1: Fig. S10). The X-ray diffraction (XRD) patterns indicated that all of the obtained nanoparticles were with a highly crystalline hexagonal phase (JCPSD28-1129, Fig. 1h). The high-resolution (HR) TEM image shows clear lattice fringes, indicating the good crystallinity of the UCNPs. The d-spacing were measured to be 0.21 nm, which were related to the (201) plane of UCNP nanocrystals (Additional file 1: Fig. S11). The EDS data and the corresponding elemental mapping of OA-UCNPs further verified that the composition of OA-UCNPs was consistent with our design (Fig. 1i and Additional file 1: Fig. S12)). Under 808-nm laser irradiation, the as-obtained UCNPs emitted around 450 nm, 478 nm and 650 nm, which overlapped with the absorption of 3HBQ and photosensitizer Ce6, respectively (Fig. 1j), implying that the specifically designed UCNPs would be able to function as photon transducers for NIR light-driven CO therapy and PDT. It is also worth noting that because of the co-doping of Yb and Tm in the core, the UCNPs also can be excited with 980-nm light yielding an emission at 800 nm (Additional file 1: Fig. S13), which can be employed to track the therapeutic nanoplatform by upconversion luminescence (UCL) imaging in vivo. To endow UCNPs with water dispersibility, an amphiphilic molecule, phosphatidylcholine (PC), was coated on the surface of OA-UCNPs. As shown in Fourier transform infrared spectra, the characteristic bands of PC were observed in the spectrum of PC-UCNPs, which were assigned to stretching vibration of P = O group (1205 cm^−1^) and stretching vibration of P-O-C group (1090 cm^−1^), verifying the successful coating of PC molecules on the surface of the nanoparticles (Additional file 1: Fig. S14).

### Construction of PDT combined with CO therapy platform

**Fig. 2:**
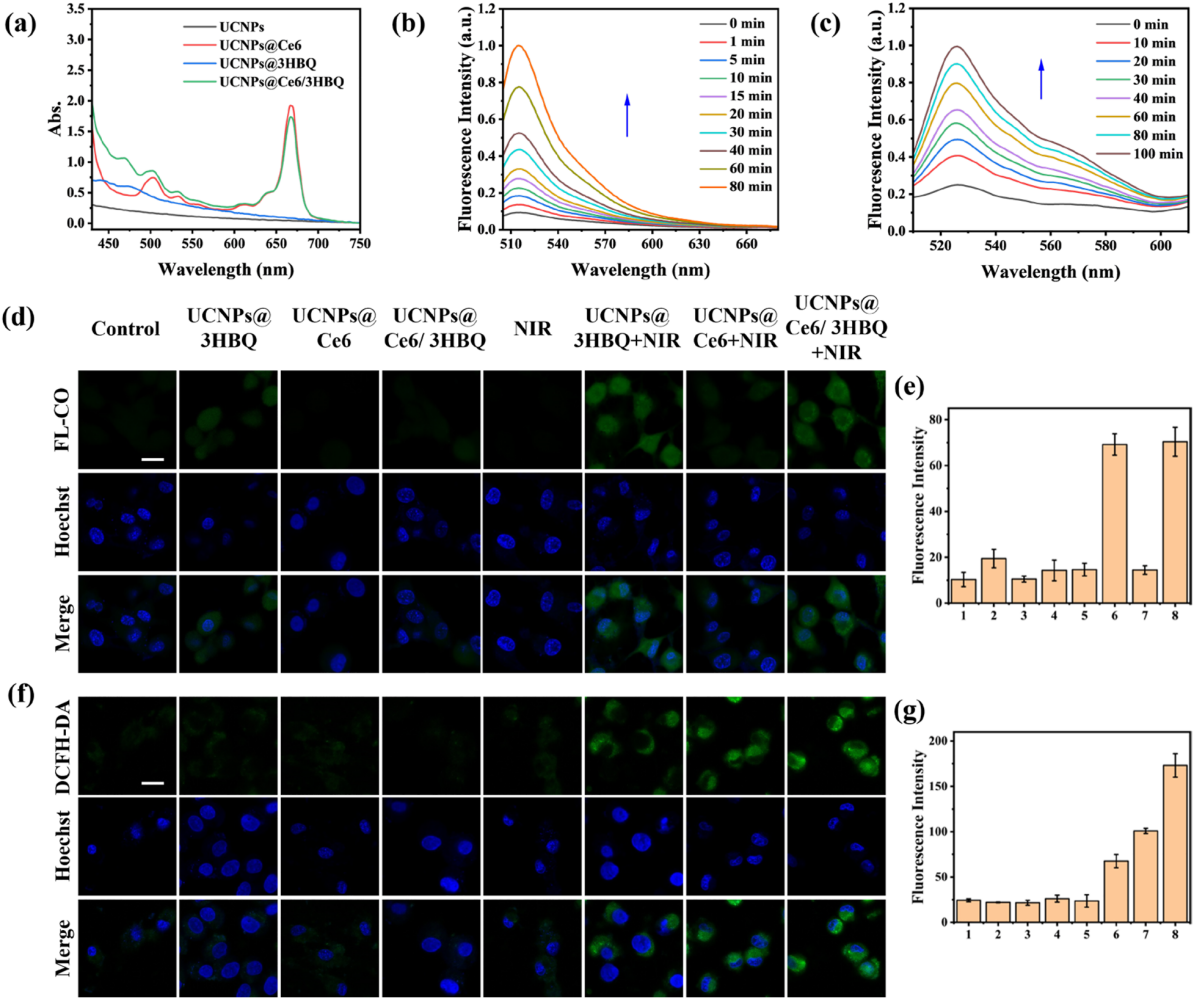
Construction of PDT combined with CO therapy platform. **a** UV–vis absorption spectra of UCNPs, UCNPs@Ce6, UCNPs@3HBQ and UCNPs@Ce6/3HBQ. **b** Fluorescence spectra of CO probe system (5 μM FL-CO + 5 μM PdCl_2_) incubated with UCNPs@Ce6/3HBQ and irradiated with 808-nm laser (0.3 W/cm^2^). **c** Fluorescence spectra of DCFH incubated with UCNPs@Ce6/3HBQ and irradiated with 808-nm laser (0.3 W/cm^2^). CLSM images of U87MG cells treated with (1) control, (2) UCNPs@3HBQ, (3) UCNPs@Ce6, (4) UCNPs@Ce6/3HBQ, (5) NIR, (6) UCNPs@3HBQ + NIR, (7) UCNPs@Ce6 + NIR and (8) UCNPs@Ce6/3HBQ + NIR after incubation with FL-CO + PdCl_2_ **d** or DCFH-DA **f** for 20 min. Nuclei were stained with Hoechst 33342. Scale bar: 20 µm. Average fluorescence intensity of FL-CO **e** and DCF **g** in U87MG cells treated with different conditions in **d** and **f**, respectively

Subsequently, we loaded 3HBQ and Ce6 onto the surface of PC-UCNPs via a hydrophobic interaction to produce UCNPs@Ce6/3HBQ. For comparison, we also constructed the nanocomposites UCNPs@Ce6 and UCNPs@3HBQ, loaded with Ce6 or 3HBQ only, respectively. The UV–vis absorption spectra were measured to verify the successful fabrication of these nanocomposites. As shown in Fig. 2a, the spectra of UCNPs@Ce6 and UCNPs@3HBQ exhibited an absorption peak around 650 nm or 478 nm, respectively. While upon the assembly with both 3HBQ and Ce6, UCNPs@Ce6/3HBQ displayed absorption peaks both around 478 nm and 650 nm. In addition, this modification step had no obvious effect on the size and morphology of UCNPs (Additional file 1: Fig. S15). According to the absorbance of UCNPs@Ce6/3HBQ at 478 nm and 650 nm as well as the molar absorption coefficients of 3HBQ and Ce6, the loading capacities of 3HBQ and Ce6 were calculated to be 83.2 and 44.2 nmol/mg PC-UCNPs, respectively (Fig. 2a and Additional file 1: Fig. S16). In order to investigate whether Ce6 and 3HBQ loaded on UCNPs surface were able to leak over time, we measured the UV–visible absorption spectra of UCNPs@Ce6/3HBQ and the supernatant after centrifugation at different time points, and found that there was no significant change of these spectra within 48 h, indicating that Ce6 and 3HBQ were stably loaded on the surface of UCNPs (Additional file 1: Fig. S17).

With the nanocomposites in hand, we estimated their capability of generating CO and ROS. After irradiating the UCNPs@Ce6/3HBQ solution containing FL-CO with an 808-nm laser, the fluorescence intensity of FL-CO was gradually enhanced with the increased irradiation time, indicating the light-induced release of CO (Fig. 2b). To examine the ability of UCNPs@Ce6/3HBQ to generate ROS, 2,7-dichlorofluorescein diacetate (DCFH) probe was incubated with the nanocomposites and irradiated with 808-nm laser. Because of the transformation of non-fluorescent DCFH to fluorescent DCF, the fluorescence intensity of DCF at 525 nm was elevated as prolonging the irradiation time, demonstrating that ROS was produced by UCNPs@Ce6/3HBQ under NIR irradiation (Fig. 2c). As shown in Additional file 1: Fig. S18, the control groups, UCNPs@3HBQ and UCNPs@Ce6, were also able to generate CO and ROS, respectively. To exclude the direct effect of NIR on 3HBQ and Ce6 molecules, they were irradiated with 808-nm laser for 10 min and detected with UV–vis absorption and fluorescence spectroscopy. As shown in Additional file 1: Fig. S19a, b, there was no significant change in the UV–vis absorption spectra of 3HBQ or emission spectra of DCFH. In addition, fluorescence emission of DCFH incubated with PC-UCNPs also kept unchanged after being irradiated with 808-nm laser (Additional file 1: Fig. S19c). All the above results revealed that neither 3HBQ nor Ce6 can be directly activated by 808-nm light, and that UCNPs was able to absorb the 808-nm photons and transfer their energy to activate 3HBQ and Ce6 to release CO and ROS, respectively.

Before studying the generation of CO and ROS at the cellular level, stability of UCNPs@Ce6/3HBQ in different media was investigated. The hydrodynamic diameter of UCNPs@Ce6/3HBQ hardly changed in HEPES buffer during 7 days (Additional file 1: Fig. S20a). The solution of UCNPs@Ce6/3HBQ was also uniform and stable during this time (Additional file 1: Fig. S20b). Meanwhile, the UCL at 450 nm of UCNPs@Ce6/3HBQ in different media including HEPES buffer (pH = 7.4, 10 mM), DMEM, 10% FBS, and 20-fold diluted whole blood kept stable within 7 days (Additional file 1: Fig. S20c), confirming the excellent thermodynamic and chemical stability of the as-obtained nanocomposite. The cellular uptake of the nanocomposites was then assessed by incubating U87MG cells with UCNPs@Ce6/3HBQ for different time. Confocal fluorescence scanning microscopy (CLSM) images showed that the intracellular UCL signal gradually increased and reached a maximum value at 4 h (Additional file 1: Fig. S21), indicating that UCNPs@Ce6/3HBQ can be efficiently endocytosed into cells within 4 h. Thus, the incubation time in the follow-up experiments was set at 4 h. Thereafter, we investigated the ability of the nanocomposites to release CO and generate ROS in living cells. FL-CO was still applied to detect CO in cells. CLSM imaging revealed that U87MG cells in the control group (no nanocomposite loaded) showed almost no fluorescence, while cells incubated with UCNPs@3HBQ or UCNPs@Ce6/3HBQ showed bright fluorescence signals under NIR light irradiation (Fig. 2d, e). DCFH-DA, a cell-permeable probe was utilized for the detection of intracellular ROS, which is non-fluorescent and can be oxidized to form fluorescent DCF by intracellular ROS [45]. As shown in Fig. 2f, g, the green fluorescence signals of DCF were extremely weak in the control groups, which were untreated, or irradiated with 808-nm laser only, or treated with nanocomposites without 808-nm laser irradiation. On the contrary, U87MG cells incubated with various nanocomposites and then irradiated with 808-nm laser exhibited strong fluorescence signals, verifying the effective generation of ROS by the nanocomposites triggered by 808-nm photons. Notably, cells treated with UCNPs@3HBQ + NIR can also emit obvious green fluorescence, suggesting that the released CO was able to cause the overproduction of intracellular ROS. As a consequence, the fluorescence intensity of cells in UCNPs@Ce6/3HBQ + NIR group was significantly stronger than that in UCNPs@Ce6 + NIR group.

### In vitro assessment of therapeutic efficiency

**Fig. 3:**
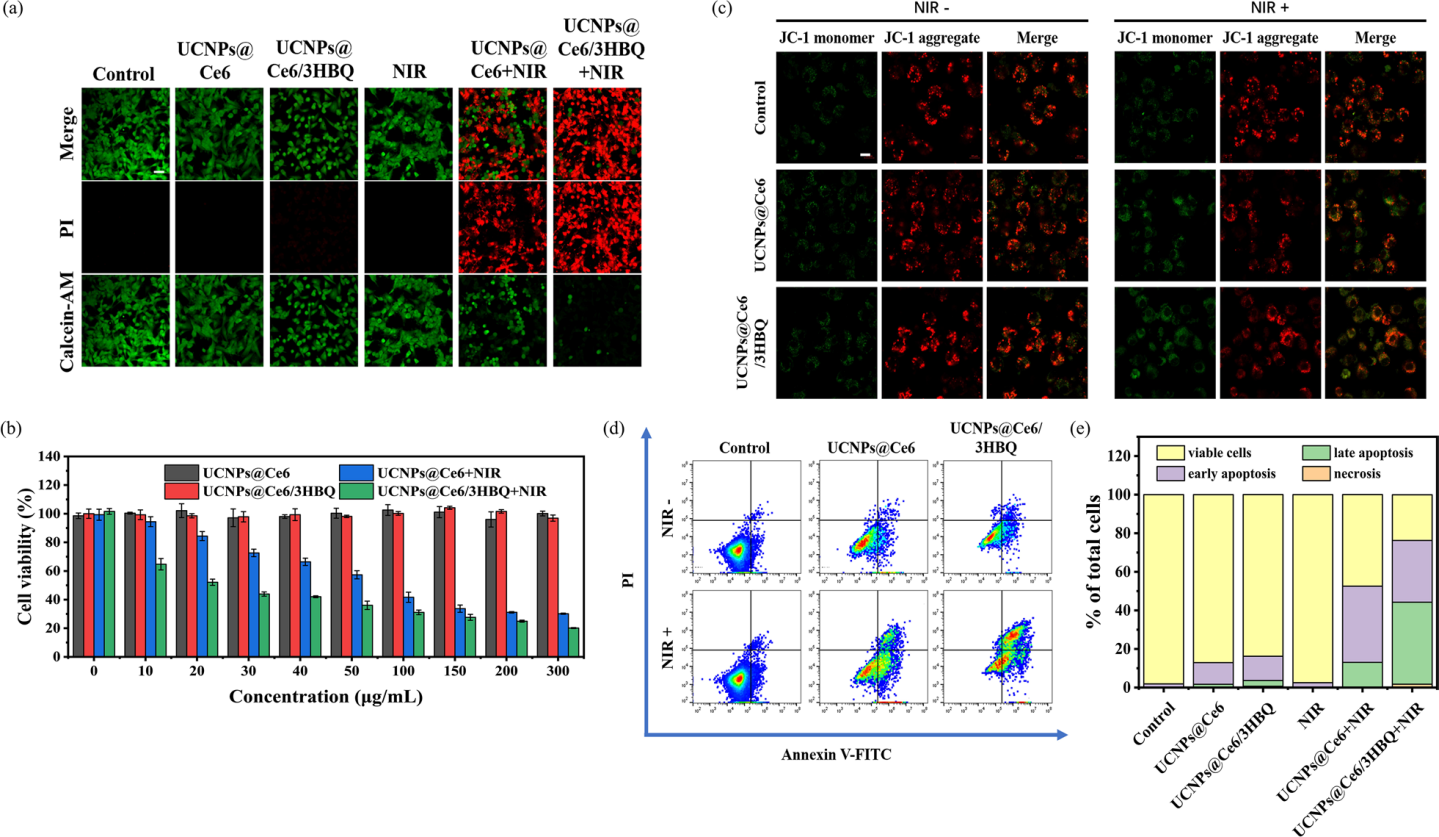
In vitro assessment of therapeutic efficiency. **a** Calcein-AM/PI staining assay of U87MG cells treated with UCNPs@Ce6/3HBQ or UCNPs@Ce6 and irradiated with or without 808-nm laser (0.8 W/cm^2^, 5 min). Scale bar: 50 μm. **b** Cell viabilities of U87MG cells after incubated with different concentrations of UCNPs@Ce6 or UCNPs@Ce6/3HBQ and irradiated with or without 808-nm laser (0.8 W/cm^2^, 5 min). **c** JC-1 staining of U87MG cells treated with UCNPs@Ce6 or UCNPs@Ce6/3HBQ and irradiated with or without 808-nm laser (0.8 W/cm^2^, 5 min). Scale bar: 20 μm. **d** Apoptosis analysis of U87MG cells after different treatments by flow cytometry. **e** Percentage of viable cells, early apoptosis, late apoptosis and necrosis obtained from **d**

To compare the ability of the UCNPs-based nanocomposites to kill tumor cells, six groups of U87MG cells were treated with different conditions, and then were stained with Calcein-AM/propidium iodide (PI) and imaged by confocal fluorescence microscopy. Calcein-AM can penetrate the membrane of living cell to stain it with a green fluorescence, while PI stains the nucleus of dead cells with a red fluorescence. Herein, UCNPs@Ce6 and UCNPs@Ce6/3HBQ were selected for comparative study in order to reveal the sensitization effect of CO on PDT. As shown in Fig. 3a, cells treated with nanocomposites only or 808-nm laser only exhibited bright green fluorescence and negligible red fluorescence, which was close to the control group of untreated cells, indicating that only 808-nm laser irradiation or nanocomposite themselves was not able to induce obvious cell death. However, cells incubated with UCNPs@Ce6 or UCNPs@Ce6/3HBQ followed by 808-nm laser irradiation showed a greatly weakened green fluorescence signal and brighter red fluorescence signal. Moreover, the number of viable cells in UCNPs@Ce6/3HBQ + NIR treated group was distinctly less than that of UCNPs@Ce6 + NIR treated group. Furthermore, cell viability under different conditions was evaluated by MTT assay. As shown in Fig. 3b, the cell viability was higher than 90% after treatment of 808-nm laser irradiation only or nanocomposites only. When cells were pretreated with UCNPs@Ce6 or UCNPs@Ce6/3HBQ for 4 h and irradiated with 808-nm laser, the cell viability decreased gradually with the concentration of the nanocomposites increasing. Also, UCNPs@Ce6/3HBQ exhibited a stronger ability to mediate cellular apoptosis than UCNPs@Ce6, which was in accordance with the results of Calcein-AM/PI co-staining.

Since apoptosis is closely related to mitochondrial dysfunction, JC-1 assay was conducted to study the mitochondrial membrane potential of the cells in different treatment groups, which emitted red fluorescence from the aggregates in normal mitochondrial membranes but green fluorescence from monomers in damaged and depolarized mitochondrial membranes [46]. As shown in Fig. 3c, strong red fluorescence from JC-1 aggregate was detected in the control, NIR irradiation only and nanocomposites incubation only groups, indicating their negligible changes in mitochondrial membrane potential. In the groups treated with UCNPs@Ce6 or UCNPs@Ce6/3HBQ together with 808-nm laser irradiation, the green fluorescence from JC-1 monomer was distinctly enhanced, and the highest ratio of green-to-red fluorescence was observed in the UCNPs@Ce6/3HBQ + NIR treated group. These results proved that both UCNPs@Ce6 and UCNPs@Ce6/3HBQ can influence the mitochondrial membrane potential under 808-nm laser irradiation, and that more serious damage to mitochondria was caused by UCNPs@Ce6/3HBQ than UCNPs@Ce6. Furthermore, to quantitatively estimate the cell fatality rate, U87MG cells were tested by flow cytometry. As shown in Fig. 3d, e, under 808-nm laser irradiation, UCNPs@Ce6/3HBQ induced apoptosis of 74.5% cells (consisting of early and late apoptosis), which was significantly higher than that induced by UCNPs@Ce6 (52.3%) under the same conditions. All the above results confirmed that the therapeutic efficiency of UCNPs@Ce6/3HBQ at the cellular level was considerably higher than that of UCNPs@Ce6, revealing that the released CO can sensitize the PDT efficiency.

Afterwards, we investigated the mechanism of improving therapeutic efficiency by the released CO. According to the previous report, CO may act on the mitochondrial electron transport chain and cause accelerated cellular respiration, leading to generation of ROS and depletion of ATP [47]. We thus measured the expression levels of several mitochondria associated proteins including heme oxygenase-1 (HMOX-1), nuclear respiratory factor-2 (NRF)-2, and serine-threonine protein kinase-1 (AKT-1) by western blot assay, all of which were obviously upregulated in CO released group (Additional file 1: Fig. S22), verifying that CO released by 3HBQ influenced mitochondria respiration. As a consequence, the level of ROS in cells was obviously improved in accordance with the result in Fig. 2f. Additionally, decrease of ATP was also found in both UCNPs@3HBQ + NIR and UCNPs@Ce6/3HBQ + NIR treated groups (Additional file 1: Fig. S23). Besides, to verfiy the inflammatory reactions can be reduced owing to the presence of CO, we detected the expression levels of proinflammatory cytokines tumor necrosis factor-α (TNF-α) and interleukin-6 (IL-6). As expected, the treatment of UCNPs@Ce6 + NIR dramatically increased the expression levels of TNF-α and IL-6, indicating the remarkable pro-inflammatory response caused by PDT (Additional file 1: Fig. S24). On the contrary, the treatment of UCNPs@3HBQ + NIR displayed negligible effect on the secretion of TNF-α and IL-6. Meanwhile, the expression levels of TNF-α and IL-6 in UCNPs@Ce6/3HBQ + NIR treated cells were significantly lower than that in UCNPs@Ce6 + NIR treated cells, disclosing the released CO can effectively inhibite the inflammatory responses caused by PDT.

### In vivo therapeutic efficiency of UCNPs@Ce6/3HBQ@CM

The efficiency of drug enriched at the tumor site is one of the key factors to affect the therapeutic effect. In order to endow UCNPs@Ce6/3HBQ with GBM targeting ability, a layer of U87MG cell membrane (CM) was wrapped on the surface of the nanocomposites (termed as UCNPs@Ce6/3HBQ@CM). The zeta potentials and hydrodynamic diameter of the nanocomposites were measured by dynamic light scattering (DLS) to characterize UCNPs@Ce6/3HBQ@CM. Compared with UCNPs@Ce6/3HBQ, the zeta potential of UCNPs@Ce6/3HBQ@ CM decreased from -24.1 ± 2.2 to − 32.9 ± 1.0 mV, which was close to that of cell membranes (− 32.5 ± 1.4 mV) (Additional file 1: Fig. S25). The hydrodynamic diameter of the nanocomposite increased from 142 to 164 nm after CM coating (Additional file 1: Fig. S26). In addition, the TEM images showed the CM layer around the nanocomposite with a thickness of ~ 5.2 nm (Additional file 1: Fig. S27). By weighing the mass of UCNPs@Ce6/3HBQ before and after wrapping U87MG cell membrane, the mass percentage of the cell membrane in UCNPs@Ce6/3HBQ@CM was 37.5%. Sodium dodecyl sulfate–polyacrylamide gel electrophoresis (SDS-PAGE) was used to study the protein ingredients of nanocomposites. Similar protein bands in U87MG cell membrane and UCNPs@Ce6/3HBQ@CM were observed (Additional file 1: Fig. S28a). Cellular biomarkers, including cell membrane marker (Na^+^/K^+^ ATPase), nuclear marker (histone H3), cytoplasmic marker (GAPDH), and mitochondrial marker (cytochrome C) were detected by western blot analysis. Compared with intact U87MG cell, its cell membrane and UCNPs@Ce6/3HBQ@CM showed no other intracellular biomarkers, but clearly displayed cell membrane biomarker (Additional file 1: Fig. S28b). These results verified the successful retention of cell membrane proteins on the surface of nanoparticles. Tumor cell membrane has a variety of membrane proteins, such as N-cadherin, CD44, CD47 and galectin-3. These membrane proteins have homologous adhesion domain and take effects on the homotypic interactions among tumor cells [48, 49]. According to the western blot analysis, these crucial membrane proteins were found in the UCNPs@Ce6/3HBQ@CM, suggesting that UCNPs@Ce6/3HBQ@CM inherited all these marker proteins and corresponding properties from source cells, which may help UCNPs@Ce6/3HBQ@CM escape from phagocytosis by macrophages, cross blood–brain barrier (BBB) and target to homologous tumor cells (Additional file 1: Fig. S29a). In addition, the results of TEM images showed that U87MG cell can uptake more nanocomposites with the help of cell membrane (Additional file 1: Fig. S29b, c).

**Fig. 4:**
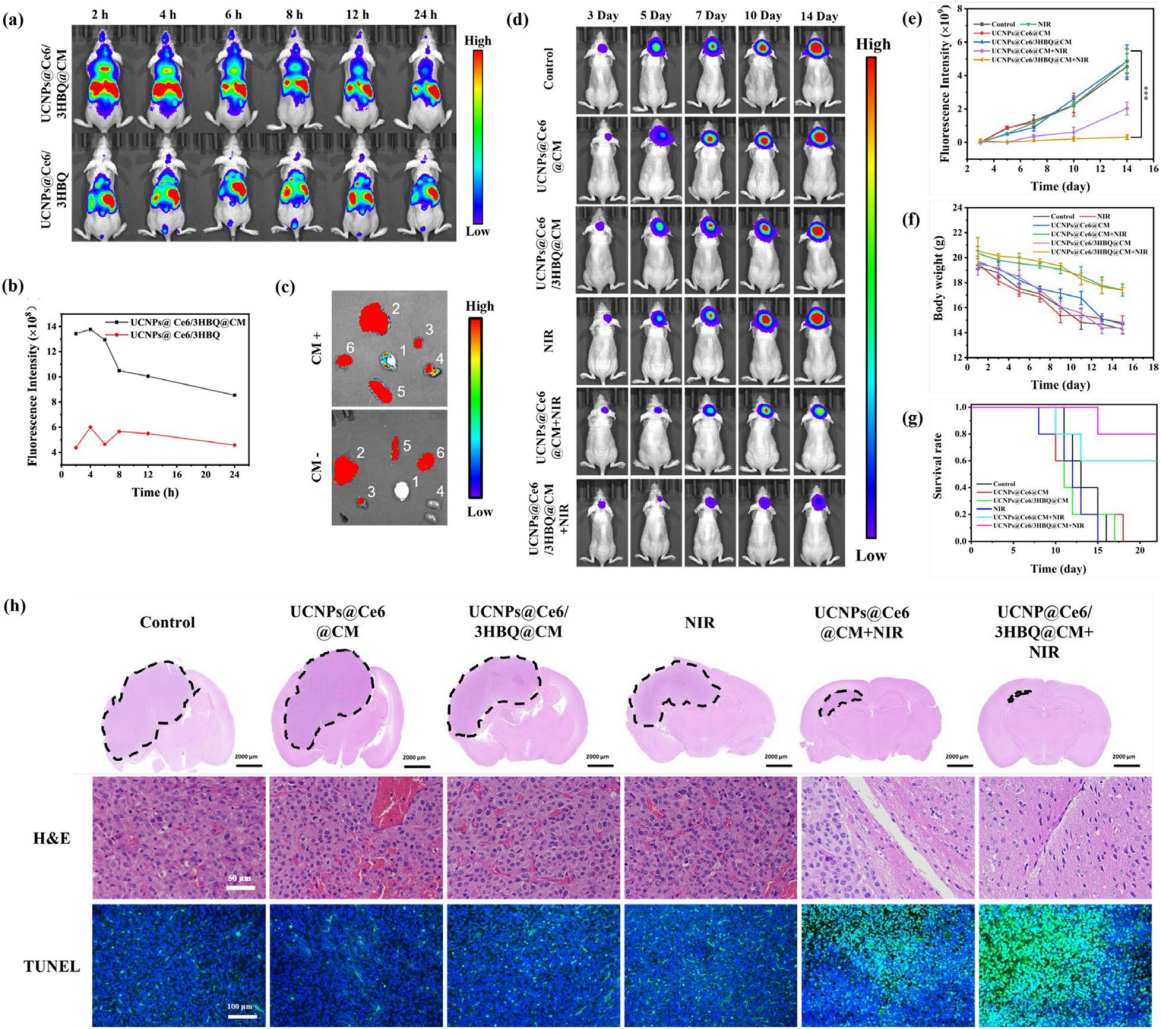
In vivo therapeutic efficiency of UCNPs@Ce6/3HBQ@CM. **a** UCL images of tumor-bearing mice after intravenous injection with the same dose of UCNPs@Ce6/3HBQ or UCNPs@Ce6/3HBQ@CM. **b** Quantitative analysis of UCL intensity in the brain at different times after injection of the two nanocomposites. **c** Ex vivo UCL images of the brain and major organs of tumor-bearing mice at 4 h after injection of the two nanocomposites. 1-Brain, 2-Liver, 3-Heart, 4-Kidney, 5-Spleen, 6-Lung. **d** Bioluminescence images of U87MG-Luc glioma-bearing mice treated with different conditions. **e** Quantitative bioluminescence intensity in the brain of the mice in **d**. **f** Body weight of U87MG-Luc glioma-bearing mice after receiving different treatments. **g** Kaplan–Meier survival curve of U87MG-Luc glioma-bearing mice with different treatments. Data are presented as mean ± SD (n ≥ 3). *P < 0.05, **P < 0.01, and ***P < 0.001. (h) Whole brain H&E staining of tumor-bearing mice treated with different conditions, and the dotted line showed the tumor area. H&E staining and TUNEL staining images of the tumor area

The biosafety of UCNPs@Ce6/3HBQ@CM was evaluated before in vivo therapy of GBM. To this end, healthy mice were randomly divided into three groups and intravenously (i.v.) injected with different dosages of UCNPs@Ce6/3HBQ@CM via tail vein (0, 75 and 150 mg/kg body weight). After 15 days of feeding, Hematoxylin–Eosin (H&E) staining was performed on the major organs including heart, liver, spleen, lung and kidney. As shown in Additional file 1: Fig. S30, there was no significant organ damage found in the mice of the three groups. Meanwhile, blood biochemical and routine analysis of the mice in the three groups exhibited similar results (Additional file 1: Fig. S31). Then the blood circulation and biological distribution of UCNPs@Ce6/3HBQ@CM were studied (Additional file 1: Fig. S32, S33). Major organs of mice were collected after injection of 7 and 14 days, and Y^3+^ content was quantified by inductively coupled plasma mass spectrometry (ICP-MS) to investigate the long-term biological distribution of UCNPs@Ce6/3HBQ@CM. ICP analysis showed that after 7 days or 14 days of injection, UCNPs@Ce6/3HBQ@CM mainly distributed in the liver and spleen and was gradually metabolized over time (Additional file 1: Fig. S33). These results suggested that UCNPs@Ce6/3HBQ@CM possessed acceptable biosafety and was suitable for in vivo application. Before studying tumor inhibition performance of UCNPs@Ce6/3HBQ@CM in vivo, we first examined whether it can be enriched in GBM by detecting the UCL signal at 800 nm under 980-nm laser excitation in the brain at varying times of post-injection (p.i.). The same dosage (75 mg/kg) of UCNPs@Ce6/3HBQ and UCNPs@Ce6/3HBQ@CM were i.v. injected into U87MG-bearing mice via the tail vein, respectively. From UCL imaging, we observed that UCL signal of the UCNPs@Ce6/3HBQ@CM group appeared in the GBM area at 2 h p.i., reached a maximum after 4 h and subsequently faded stepwise (Fig. 4a, b). In contrast, UCL signals in the GBM region of the mice injected with UCNPs@Ce6/3HBQ were obviously weaker than that of mice injected with UCNPs@Ce6/3HBQ@CM at all the tested time points. Furthermore, in vitro imaging of the brains and other major organs (heart, liver, spleen, lung, and kidney) at 4 h p.i. discovered that the brain of mice injected with UCNPs@Ce6/3HBQ@CM showed significant stronger UCL signals than the mice injected with UCNPs@Ce6/3HBQ (Fig. 4c). These results can be explained that the U87MG cell membrane encapsulation improved the accumulation and prolonged retention of the therapeutic agent in GBM because of the homologous targeting ability.

Encouraged by the above discovered in vitro anti-cancer ability of UCNPs@Ce6/3HBQ with the assistance of CO and the tumor accumulation contributed by U87MG CM coating, we further explored the tumor inhibition efficiency in vivo. The U87MG-Luc (luciferase-expressing U87MG cells) tumor-bearing mice were randomly divided into six groups and subjected to different treatments. The first group was the control group injected with saline; the second group was irradiated with 808-nm laser only; the third/fourth groups were i.v. injected with UCNPs@Ce6/3HBQ@CM or UCNPs@Ce6@CM, respectively, but without NIR irradiation; the other two groups of mice were i.v. injected with UCNPs@Ce6/3HBQ@CM or UCNPs@Ce6@CM, respectively, and irradiated with 808-nm laser. The activity of glioma cell can be detected by bioluminescence signal through injection of luciferin, thus estimating the tumor size [50]. As shown in Fig. 4d, e), the bioluminescence signal in the brain of mice treated with 808-nm laser only or nanocomposites only was similar to the control group, indicating 808-nm laser or nanocomposites themselves had no obvious effect on GBM. As expected, the bioluminescence signals were distinctly weakened in the groups treated with the nanocomposites together with 808-nm laser irradiation. In consistence with the in vitro experiments, UCNPs@Ce6/3HBQ@CM exhibited the highest *in vivo* tumor inhibition efficiency. During the therapy process, the body weight of the mice in six groups was recorded every 2 days until 15 days after treatment, which showed no significant difference among the six groups (Fig. 4f), again evidencing the good biosafety of the developed therapeutic platform. The survival curve showed that the median survival time of mice in UCNPs@Ce6/3HBQ@CM + NIR group was more than 24 days, which was significantly longer than that of control group (13 days), UCNPs@Ce6@CM group (12 days), UCNPs@Ce6/3HBQ@CM group (12 days), NIR (12 days) and UCNPs@Ce6@CM + NIR group (18 days) (Fig. 4g). As a further proof of the therapeutic effect, H&E and TUNEL staining was conducted to observe the morphology of organs from GBM site. The results showed that UCNPs@Ce6/3HBQ@CM can induce extensive necrosis and apoptosis of GBM tissue and offer the highest therapeutic efficiency (Fig. 4h). Moreover, similar to the in vitro results, the intratumoral HMOX-1 and NRF-2 levels in mice treated with UCNPs@Ce6/3HBQ@CM + NIR were also found to be upregulated, further suggesting the influence of the in situ released CO on mitochondrial function of tumor cell, which thereby enhanced therapeutic efficacy of GBM treatment (Additional file 1: Fig. S34). Besides, the expression levels of TNF-α and IL-6 were minimally affected by UCNPs@Ce6/3HBQ@CM + NIR, further suggesting the inflammatory responses caused by PDT can be also restrained by CO in vivo (Additional file 1: Fig. S35). In addition, H&E staining of other major organs of the mice on the 15th day after treatment showed little difference among the six groups, implying all of the treatments had ignorable adverse effect on the mice (Additional file 1: Fig. S36). Taken together, the above in vivo investigations firmly established the improved therapeutic efficacy through releasing CO at the tumor site.

## Conclusions

In summary, we successfully constructed a NIR light-triggered therapeutic nanoplatform with combination of PDT and CO for enhanced GBM treatment, in which CO and ROS can be simultaneously released by an upconverted transducer. This nanocomposite was composed of UCNPs with multi-color emission, photoCORM 3HBQ and photosensitizer Ce6. Under a highly penetrable 808-nm laser irradiation, UCNPs emitted blue light to trigger the release of CO and red light to activate Ce6 to generate ROS, respectively. CO released in situ can interfere with mitochondrial respiration and deplete ATP in tumor cells as well as reduce the inflammatory responses caused by PDT, thus effectively enhancing the rate of tumor cell apoptosis and the inhibition of tumor growth and alleviating side effects. By encapsulating the nanocomposites with U87MG cell membranes, the therapeutic agent was able to target GBM and enrich in the tumor site via homologous targeting, achieving the precise and efficient treatment of intractable GBM.

## Experimental

### Materials and instruments

LnCl_3_, oleic acid, 1-octadecene and chlorin e6 (Ce6) were purchased from Aladdin Reagent, Ltd. (Shanghai, China). DSPE-PEG was provided by Xi'an ruixi Biological Technology Co., Ltd. Calcein-AM and Propidium Iodide (PI) were supplied by Sigma Aldrich. Other chemical reagents were obtained from Sinopharm Chemical Reagent Co., Ltd. (Shanghai, China). All chemical reagents were analytical grade or higher and used without further purification. All aqueous solutions were prepared with ultrapure water (Mill-Q, Millipore, 18.2 MΩ resistivity). Female Balb/c nude mice and female Balb/c mice (~ 20 g) were purchased from Hubei Beiente Biotechnology Co., Ltd. The crystal phase was acquired by using an X-ray diffractometer (XRD, Bruker D8 Discover) with a 2θ range of 10° − 80° with Cu Kα irradiation (k = 1.5406 Å). The UCL spectra were recorded on an RF-5301 fluorophotometer (Shimadzu, Japan) with an external 808-nm CW laser as the excitation source. The morphology and size of UCNPs were characterized by a JEM-2010 transmission electron microscope (TEM) operated at 200 kV. The FTIR spectra were obtained by a Nicolet iS10 FTIR Spectrometer (Thermo Fisher Scientific, USA) with the KBr pellet technique. UV–vis absorption spectra were acquired from a UH-4150 spectrophotometer (Hitachi, Japan). CCK-8 test was acquired by Multiskan GO microplate reader (Thermo Scientific Multiskan, USA). Fluorescence microscopy images of U87MG cells were conducted on Zeiss LSM 880 Microscope. In vivo UCL imaging was conducted with Perkin Elmer IVIS Spectrum.

### Synthesis of 3HBQ

3HBQ was prepared as reported [42].

### Synthesis of oleic acid-modified upconversion nanoparticles (OA-UCNPs)

OA-stabilized NaYF_4_:Yb/Tm@NaYF_4_:Nd@NaYF_4_@NaErF_4_:Tm@NaYF_4_ nanoparticles were synthesized through a seed-mediated epitaxial growth method described in our previous work [51]. First, 0.75 mmol Ln(oleate)_3_ (Y^3+^:Yb^3+^:Tm^3+^  = 79.5:20:0.5) and 20 mmol NaF were added into 20 mL of the mixture solvent, which contained the equal volumes of oleic acid (OA) and 1-octadecene (ODE) in a three-neck flask. After vacuuming, the mixture was kept at 110 °C for 1 h in an argon atmosphere. The mixture was then heated to 320 °C and kept for 2.5 h to gain the core NaYF_4_:Yb/Tm. The S1 layer was then grown on the core by injection of 0.2 mmol Ln(oleate)_3_ (Ln = Y^3+^:Nd^3+^  = 70:30) and maintained the reaction for another 40 min. The S2 layer was then grown on NaYF_4_:Yb/Tm@NaYF_4_:Nd by injection of 0.4 mmol Y(oleate)_3_ and maintained the reaction for another 40 min. The S3 layer was then grown on NaYF_4_:Yb/Tm@ NaYF_4_:Nd@NaYF_4_ by injection of 0.6 mmol Ln(oleate)_3_ (Ln = Er^3+^:Tm^3+^  = 99.5:0.5) and maintained the reaction for another 60 min. The S4 layer was then grown on NaYF_4_:Yb/Tm@NaYF_4_:Nd@NaYF_4_@NaErF_4_:Tm by injection of 0.4 mmol Y(oleate)_3_ and maintained the reaction for another 40 min. After the temperature cooled to room temperature, the mixture was precipitated by adding equal volume of ethanol. The nanoparticles were centrifuged and washed three times with hexane/ethanol (v/v = 1:1). Finally, the obtained OA-UCNPs were dispersed in hexane for standby.

### Loading Ce6/3HBQ on the surface of UCNPs

OA-UCNPs (1 mg/mL) and PC (1 mg/mL) in CHCl_3_ were mixed with a volume ratio of 1:2. The solvent was evaporated under a nitrogen atmosphere, and then the solid was dispersed in water with a concentration of 1 mg/mL. Different volumes of Ce6/3HBQ (10 mM in DMSO) were added into the mixture. After vigorous shaking for 1 min, the mixture was centrifuged for 15 min and then washed twice with ultrapure water. The obtained complex was dispersed in ultrapure water with a final concentration of 1 mg/mL.

### Detection of CO release in solution

The generation of CO in the solution was measured by a CO probe system (FL-CO + PdCl_2_). Due to the lactone form of the fluorescein structure, FL-CO is non-fluorescent. With the conversion of Pd^2+^ to Pd^0^ by CO, the allyl group on FL-CO was released based on the well-known Pd^0^-mediated Tsuji-Trost reaction to recover the fluorescence. UCNPs@3HBQ or UCNPs@Ce6/3HBQ were added into 10 μM FL-CO and 10 μM PdCl_2_ and irradiated with an 808-nm laser (0.3 W/cm^2^). The generation of CO was determined by measuring the fluorescence spectra of FL-CO (λ_ex_ = 500 nm).

### Detection of ROS generation in solution

To verify the ROS generation ability, UCNPs@Ce6/3HBQ or UCNPs@Ce6 were added into 10 μM DCFH and irradiated with an 808-nm laser (0.3 W/cm^2^). The generated ROS were determined by measuring the fluorescence spectrum of DCF (λ_ex_ = 488 nm).

### Cell culture and establishment of animal models

U87MG (human glioblastoma cells) were incubated in MEM medium containing 10% FBS, 1% antibiotics, 1% Non-Essential Amino Acids (NEAA), and 1 mM Sodium Pyruvate (NaP). Luciferase-expressing U87MG (U87MG-Luc) were grown in MEM medium with 10% FBS, 1% antibiotics, 1% NEAA, 1 mM NaP, and 1 ug/mL puromycin. All the cells were incubated at 37 °C in a humidified incubator with 5% CO_2_.

GBM model: BALB/c nude mice were anesthetized with 2.0% isoflurane and set on a stereotactic instrument. Then, 5.0 × 10^5^ U87MG-Luc cells were injected into the right striatum (bright lateral: 2.0 mm, bregma: 0.5 mm, depth: 3.5 mm) of nude mice by using a mouse adaptor. The growth of intracranial glioblastoma was monitored by bioluminescence imaging.

### Acquisition of U87MG cell membranes

The collected U87MG cells were washed with PBS three times. Then, they were dispersed in membrane protein extraction buffer solutions and cooled in an ice bath for 15 min. Subsequently, the solution was frozen and then thawed. Such a cycle of freezing–thawing was performed three times. Subsequently, the mixture was centrifuged at 700 g for 10 min at 4 °C and the obtained cell suspensions were further centrifuged at 14,000 g for 30 min at 4 °C. The precipitate was resuspended in deionized water and stored at − 80 °C.

### Preparation of cell membrane coated nanoparticles

UCNPs@Ce6/3HBQ solution (0.5 mL, 2 mg/mL) and the cell membranes solution (0.5 mL, 2 mg/mL) were mixed and sonicated for 10 min to obtain the cell membrane coated nanoparticles.

### In vitro cellular uptake of nanocomposites

The cellular uptake process was investigated by confocal laser scanning microscopy (CLSM) on U87MG cells. Typically, U87MG cells were seeded into dishes with a glass bottom and cultured in an atmosphere of 5/95 (v/v) of CO_2_/air at 37 °C for 24 h. Then, the cells were incubated with a culture medium containing 0.3 mg/mL UCNPs@Ce6/3HBQ for 0, 2, 4, and 8 h. The cells were washed with PBS three times and used for CLSM imaging.

### Imaging of CO and ROS in U87MG cell

U87MG cells in 35 mm glass dishes were treated with (1) PBS only, (2) UCNPs@3HBQ (300 μg/mL) only, (3) UCNPs@Ce6 (300 μg/mL) only, (4) UCNPs@Ce6/3HBQ (300 μg/mL) only, (5) 808-nm laser irradiation only, (6) UCNPs@3HBQ (300 μg/mL) and 808-nm laser irradiation, (7) UCNPs@Ce6 (300 μg/mL) and 808-nm laser irradiation and (8) UCNPs@Ce6/3HBQ (300 μg/mL) and 808-nm laser irradiation, respectively. After incubated with nanocomposites for 4 h, the cells were exposed with an 808-nm laser for 3 min (with intermittent 30 s breaks after each 30 s of irradiation to prevent overheating) with a power density of 0.8 W/cm^2^. Then, all of the cells were incubated with a medium containing FL-CO (5 μM) + PdCl_2_ (5 μM) or DCFH-DA (10 μM) for 30 min. The cells were washed with PBS two times and used for fluorescence microscope imaging.

### In vitro anticancer efficacy

The cellular cytotoxicity was measured by a standard MTT assay. U87MG cells were seeded into 96-well plates and incubated in an atmosphere of 5/95 (v/v) of CO_2_/air at 37 °C for 24 h. Then the cells were incubated with different concentrations of UCNPs@Ce6, or UCNPs@Ce6/3HBQ (0, 10, 20, 30, 40, 50, 100, 150, 200, 300 μg/mL) for 4 h. The cells were washed with PBS two times and treated with 808-nm laser irradiation for 5 min (with intermittent 30 s breaks after each 30 s of irradiation to prevent overheating) with a power density of 0.8 W/cm^2^. After 12 h incubating under the same condition, 20 μL of MTT solution (5.0 mg/mL) was added into each well. Then, the medium was replaced by 150 μL of DMSO after 4 h incubation, and the absorbance at 490 nm was measured by a microplate reader. Cell viability was calculated by A/A_0_ × 100% (A and A_0_ are the absorbance of the experimental group and control group, respectively). Dark cytotoxicity was measured in the same manner as described above in the absence of 808-nm laser irradiation.

The cellular cytotoxicity was also investigated by Calcein-AM staining assay. U87MG cells were seeded into 35 mm glass dishes and incubated for 24 h. Subsequently, the cells were divided into six groups and treated with (1) PBS only, (2) 808-nm laser irradiation only, (3) UCNPs@Ce6 (300 μg/mL) only, (4) UCNPs@Ce6/3HBQ (300 μg/mL) only, (5) UCNPs@Ce6 (300 μg/mL) and 808-nm laser irradiation, and (6) UCNPs@Ce6 (300 μg/mL) and 808-nm laser irradiation. After incubated with nanocomposites for 4 h, the cells were exposed with an 808-nm laser for 5 min (with intermittent 30 s breaks after each 30 s of irradiation to prevent overheating) with a power density of 0.8 W/cm^2^. Then, the cells were incubated for another 12 h, and 20 μL of Calcein-AM solution was added and incubated for 20 min. The cells were washed with PBS two times and used for fluorescence microscope imaging.

### Mitochondrial membrane potential

U87MG cells were seeded into 35 mm glass dishes and incubated for 24 h. Subsequently, the cells were divided into six groups and treated with (1) PBS only, (2) 808-nm laser irradiation only, (3) UCNPs@Ce6 (300 μg/mL) only, (4) UCNPs@Ce6/3HBQ (300 μg/mL) only, (5) UCNPs@Ce6 (300 μg/mL) and 808-nm laser irradiation, and (6) UCNPs@Ce6 (300 μg/mL) and 808-nm laser irradiation. After incubated with nanocomposites for 4 h, the cells were exposed with an 808-nm laser for 5 min (with intermittent 30 s breaks after each 30 s of irradiation to prevent overheating) with a power density of 0.8 W/cm^2^. After additional incubation for 4 h, the cells were incubated with JC-1 (200 μL, 10 μg/mL) for 10 min, and washed with PBS twice. Then, the cells were detected by a CLSM. The green channel of monomer was excited by 488 nm laser, and the emission wavelength range was collected at 530 ± 15 nm. The red images of J-aggregate were excited by 514 nm laser, and the emission wavelength range was collected at 590 ± 17 nm.

### Flow cytometry assays

U87MG cells were seeded into 6-well plates and incubated for 24 h. Subsequently, the cells were treated with (1) PBS only, (2) 808-nm laser irradiation only, (3) UCNPs@Ce6 (300 μg/mL) only, (4) UCNPs@Ce6/3HBQ (300 μg/mL) only, (5) UCNPs@Ce6 (300 μg/mL) and 808-nm laser irradiation, and (6) UCNPs@Ce6 (300 μg/mL) and 808-nm laser irradiation. Both suspended and adherent cells in each group were collected and resuspended in 1.0 mL of binding buffer. After incubating with annexin-V-FITC for 10 min and PI for 5 min at 4 °C in the dark, the cells were analyzed by flow cytometry.

### In vivo toxicity

Different dosages of UCNPs@Ce6/3HBQ@CM (0, 75 mg/kg, 150 mg/kg) were injected intravenously into healthy female Kunming mice, respectively. After 15 days, major organs (heart, spleen, liver, lung, kidney) and blood were separately used for H&E staining and hematology analysis. To investigate the long-term toxicity of UCNPs@Ce6/3HBQ@CM in various organs of mice, mice were given UCNPs@Ce6/3HBQ@CM intravenously (75 mg/kg). After 7 days, the mice were sacrificed, and the major organs (heart, liver, spleen, lung, kidney) and brain were taken. Y^3+^ concentration was determined by inductively coupled plasma mass spectrometry (ICP-MS).

### Nanoparticles biodistribution in glioma-bearing mice

The U87MG intracranial orthotopic glioblastoma mice were randomly divided into two groups and administered via tail injection with UCNPs@Ce6/3HBQ (10 mg/mL, 100 μL) or UCNPs@Ce6/3HBQ@CM (10 mg/mL, 100 μL), respectively. Fluorescence images were obtained at different time points after injection of nanocomposites (2, 4, 6, 8, 12, and 24 h) with the in vivo fluorescence imaging system (IVIS Spectrum, PerkinElmer). At 4 h post-injection, mice were euthanized to collect the main organs (heart, liver, spleen, lung, kidney, and brain) for In vitro fluorescence imaging. To investigate the blood circulation, the mice were intravenously injected with UCNPs@Ce6/3HBQ@CM (75 mg/kg). At different time points post-injection, the blood samples were collected in heparinized tubes by tail clipping and was centrifuged at 3000 g for 10 min to obtain serum. The concentration of UCNPs@Ce6/3HBQ@CM in the blood is determined by up-conversion luminescence of the sample.

In vivo anticancer efficacy.

The U87MG intracranial orthotopic glioblastoma mice were randomly assigned into six groups (5 mice per group). They were treated with (1) PBS only, (2) 808 nm laser irradiation only, (3) UCNPs@Ce6@CM (10 mg/mL, 100 μL) only, (4) UCNPs@Ce6@CM (10 mg/mL, 100 μL) and 808-nm laser irradiation, (5) UCNPs@Ce6/3HBQ@CM (10 mg/mL, 100 μL) only, (6) UCNPs@Ce6/3HBQ@CM (10 mg/mL, 100 μL) and 808-nm laser irradiation. All materials were intravenously injected into the mice through the tail. After injecting the drug for 4 h, the mice were exposed to an 808-nm laser for 5 min (with intermittent 30 s breaks after each 30 s of irradiation to prevent overheating) with a power density of 0.8 W/cm^2^. Subsequently, we measured the tumor size and the body weight every 2 days. When the mice grew up to 15 days, all of them were sacrificed and dissected to collect the tumor tissues for further H&E and TUNEL staining analysis.

### Statistical analysis

Statistical significance was performed by using Student’s t-test (two-tailed), in which P < 0.05 (*), P < 0.01 (**) and P < 0.001 (***) were considered statistically significant.

## Supplementary Information

**Additional file 1: Figure S1**. ^1^H-NMR spectrum of 3HBQ. **Figure S2**. ^13^C-NMR spectrum of 3HBQ. **Figure S3**. High resolution mass spectrum (HRMS) of 3HBQ. **Figure S4**. 3HBQ solution light response releases CO. **Figure S5**. The fluorescence emission spectra of 3HBQ solution after being irradiated with a 450-nm LED light source. **Figure S6**. The mechanism of CO detecting by FL-CO. **Figure S7**. The fluorescence spectra of FL-CO incubated with 3HBQ and irradiated for different times in 50% DMSO-PBS buffer. **Figure S8**. HRMS of the product obtained by irradiating 3HBQ. **Figure S9**. The mechanism of CO release by 3HBQ. **Figure S10.** The histograms of the size distribution of (a) C, (b) C-S1, (c) C-S1-S2, (d) C-S1-S2-S3, (e) C-S1-S2-S3-S4 structured UCNPs. **Figure S11.** HRTEM image of OA-UCNPs. **Figure S12.** Constituent elements and contents of OA-UCNPs. **Figure S13**. Upconversion emission spectrum of UCNPs under 980-nm laser excitation. **Figure S14**. FTIR spectra of OA-UCNPs, PC and PC-UCNPs. **Figure S15**. The TEM image (a) and histograms of the size distribution (b) of UCNPs@Ce6/3HBQ. **Figure S16**. UV–vis absorption spectra of (a) Ce6 and (c) 3HBQ with different concentrations in DMSO. **Figure S17.** UV–visible absorption spectra of UCNPs@Ce6/3HBQ and the supernatant after centrifugation within 48 h. **Figure S18**. Detection of reactive oxygen species production by UCNPs@Ce6 and CO release by UCNPs@3HBQ. **Figure S19**. Control experiments to exclude the direct effect of NIR on 3HBQ and Ce6 molecules. **Figure S20**. UCNPs@Ce6/3HBQ stability test. **Figure S21**. UCNPs@Ce6/3HBQ cell uptake imaging. **Figure S22**. Expression levels of HMOX-1, AKT-1 and NRF-2 in U87MG cells after receiving different treatments. **Figure S23**. ATP levels in U87MG cells after receiving different treatments. **Figure S24**. Expression levels of TNF-α and IL-6 in U87MG cells after receiving different treatments. **Figure S25**. Zeta potential of 1) CM, 2) UCNPs@Ce6/3HBQ and 3) UCNPs@Ce6/3HBQ@CM. **Figure S26**. Hydrodynamic diameter of UCNPs@Ce6/3HBQ and UCNPs@Ce6/3HBQ@CM. **Figure S27**. The TEM image and histogram of the size distribution of UCNPs@Ce6/3HBQ@CM. **Figure S28.** SDS-PAGE protein analysis and western blot analysis of UCNPs@Ce6/3HBQ@CM. **Figure S29.** Western blot analysis and TEM images of UCNPs@Ce6/3HBQ@CM**. Figure S30**. H&E staining of major organs (healthy mice) after injection with different dosages of UCNPs@Ce6/3HBQ@CM. **Figure S31**. Blood routine and serum biochemical levels of mice after injection with different doses of UCNPs@Ce6/3HBQ@CM. **Figure S32.** Blood concentration–time curve of UCNPs@Ce6/3HBQ@CM in mice. **Figure S33.** Y^3+^ content in major organs of mice after injection of UCNPs@Ce6/3HBQ@CM for 7 days and 14 days. **Figure S34**. HMOX-1 and NRF-2 levels of GBM site on the 15^th^ day. **Figure S35**. TNF-α and IL-6 levels of GBM site on the 15^th^ day. **Figure S36**. H&E stained major organs of U87MG-Luc glioma-bearing mice after receiving different treatments.

## Data Availability

The datasets used and/or analyzed during the current study are available from the corresponding author on reasonable request.
