# Correction: Near-infrared light triggered in situ release of CO for enhanced therapy of glioblastoma

**DOI:** 10.1186/s12951-025-03162-y

**Published:** 2025-03-25

**Authors:** Juan Ge, Miaomiao Zuo, Qirong Wang, Zhen Li

**Affiliations:** College of Chemistry and Chemical Engineering, College of Health Science and Engineering, Hubei University, Wuhan, 430062 China

**Correction: Journal of Nanobiotechnology (2023) 21:48** 10.1186/s12951-023-01802-9

Following publication of the original article, the authors have identified errors in the figures. The images in Fig. 2d, Fig. 4d, h (tunel), and Fig. S36 were incorrectly used due to their high similarity, resulting in a mistake during the typesetting process. The authors sincerely apologize for this oversight.

The corrected images for Fig. 2d, Fig. 4d, h (tunel), and Fig. S36 are provided below and the original article has been corrected. These corrections do not affect the results or conclusions of the research.

Incorrect Fig. 2


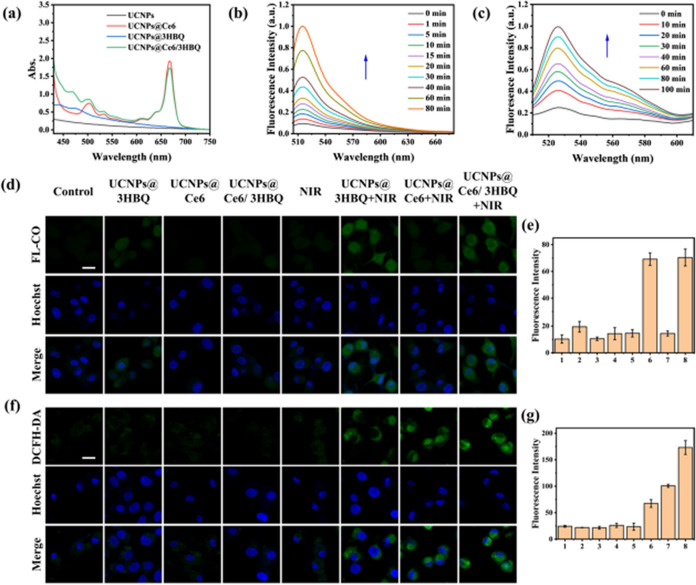


Corrected Fig. 2

**Fig. 2:**
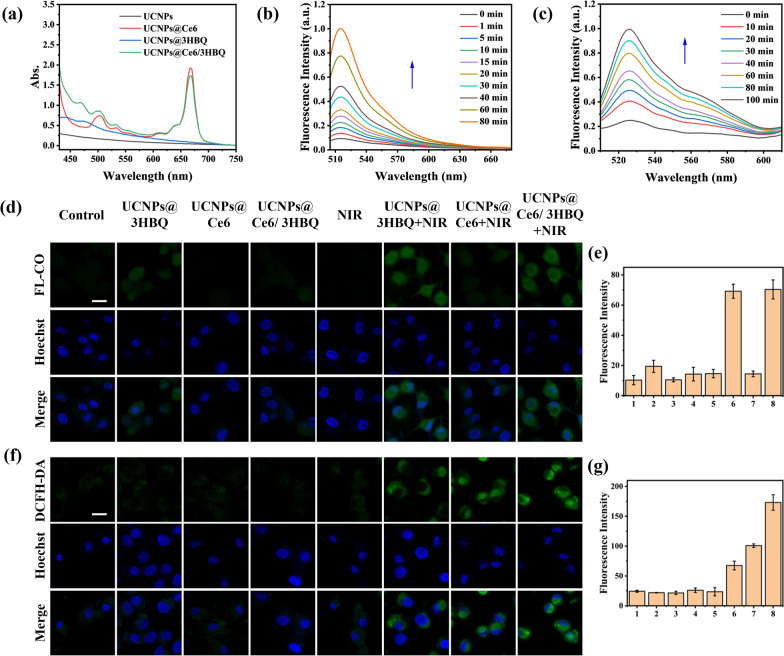
Construction of PDT combined with CO therapy platform.** a** UV–vis absorption spectra of UCNPs, UCNPs@Ce6, UCNPs@3HBQ and UCNPs@ Ce6/3HBQ.** b** Fluorescence spectra of CO probe system (5 μM FL-CO + 5 μM PdCl2) incubated with UCNPs@Ce6/3HBQ and irradiated with 808-nm laser (0.3 W/cm^2^).** c** Fluorescence spectra of DCFH incubated with UCNPs@Ce6/3HBQ and irradiated with 808-nm laser (0.3 W/cm^2^). CLSM images of U87MG cells treated with (1) control, (2) UCNPs@3HBQ, (3) UCNPs@Ce6, (4) UCNPs@Ce6/3HBQ, (5) NIR, (6) UCNPs@3HBQ + NIR, (7) UCNPs@ Ce6 + NIR and (8) UCNPs@Ce6/3HBQ + NIR after incubation with FL-CO + PdCl2 d or DCFH-DA f for 20 min. Nuclei were stained with Hoechst 33342. Scale bar: 20 μm. Average fluorescence intensity of FL-CO** e** and DCF** g** in U87MG cells treated with different conditions in** d** and** f**, respectively

Incorrect Fig. 4


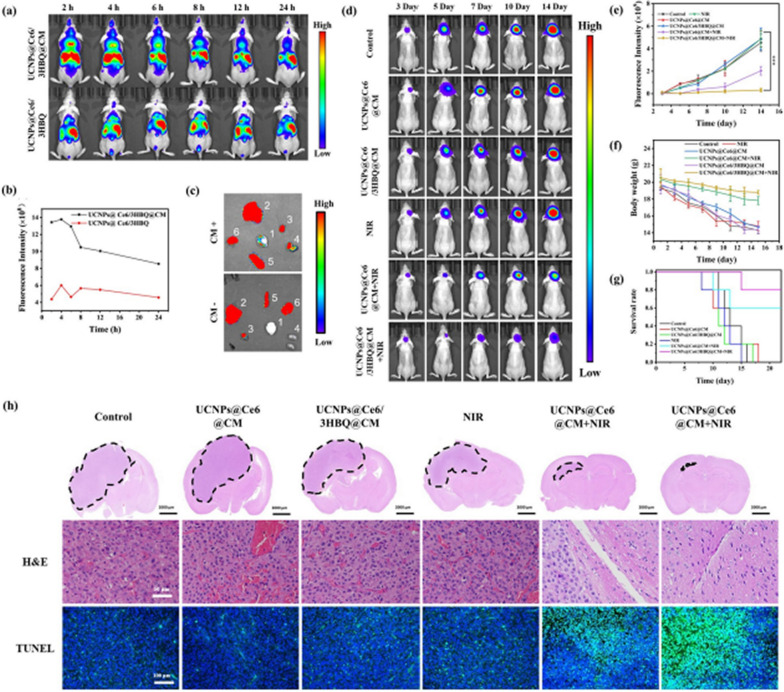


Corrected Fig. 4

**Fig. 4:**
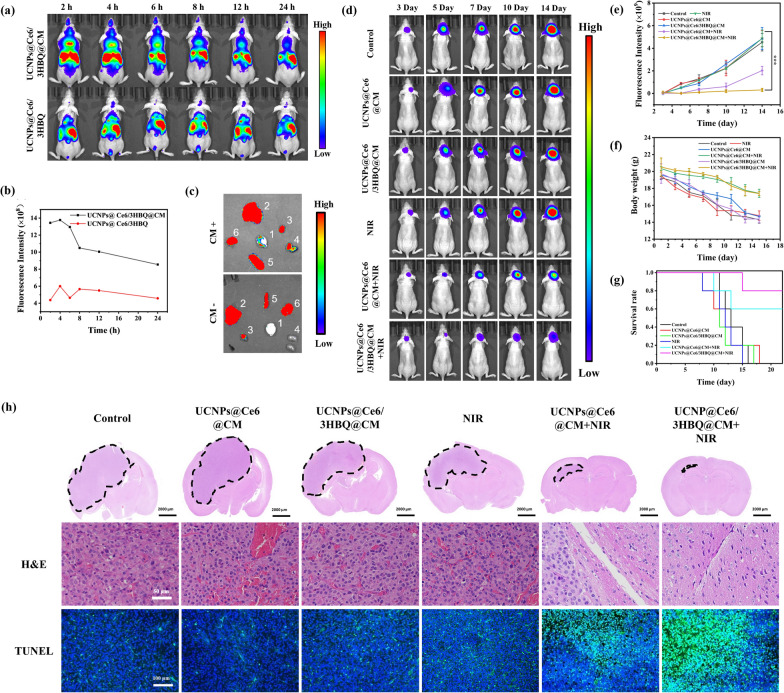
In vivo therapeutic efficiency of UCNPs@Ce6/3HBQ@CM. **a** UCL images of tumor-bearing mice after intravenous injection with the same dose of UCNPs@Ce6/3HBQ or UCNPs@Ce6/3HBQ@CM. **b** Quantitative analysis of UCL intensity in the brain at different times after injection of the two nanocomposites. **c** Ex vivo UCL images of the brain and major organs of tumor-bearing mice at 4 h after injection of the two nanocomposites. 1-Brain, 2-Liver, 3-Heart, 4-Kidney, 5-Spleen, 6-Lung. **d** Bioluminescence images of U87MG-Luc glioma-bearing mice treated with different conditions. **e** Quantitative bioluminescence intensity in the brain of the mice in **d**. **f** Body weight of U87MG-Luc glioma-bearing mice after receiving different treatments. **g** Kaplan–Meier survival curve of U87MG-Luc glioma-bearing mice with different treatments. Data are presented as mean ± SD (n ≥ 3). *P < 0.05, **P < 0.01, and ***P < 0.001. **h** Whole brain H&E staining of tumor-bearing mice treated with different conditions, and the dotted line showed the tumor area. H&E staining and TUNEL staining images of the tumor area

Incorrect Fig. S36


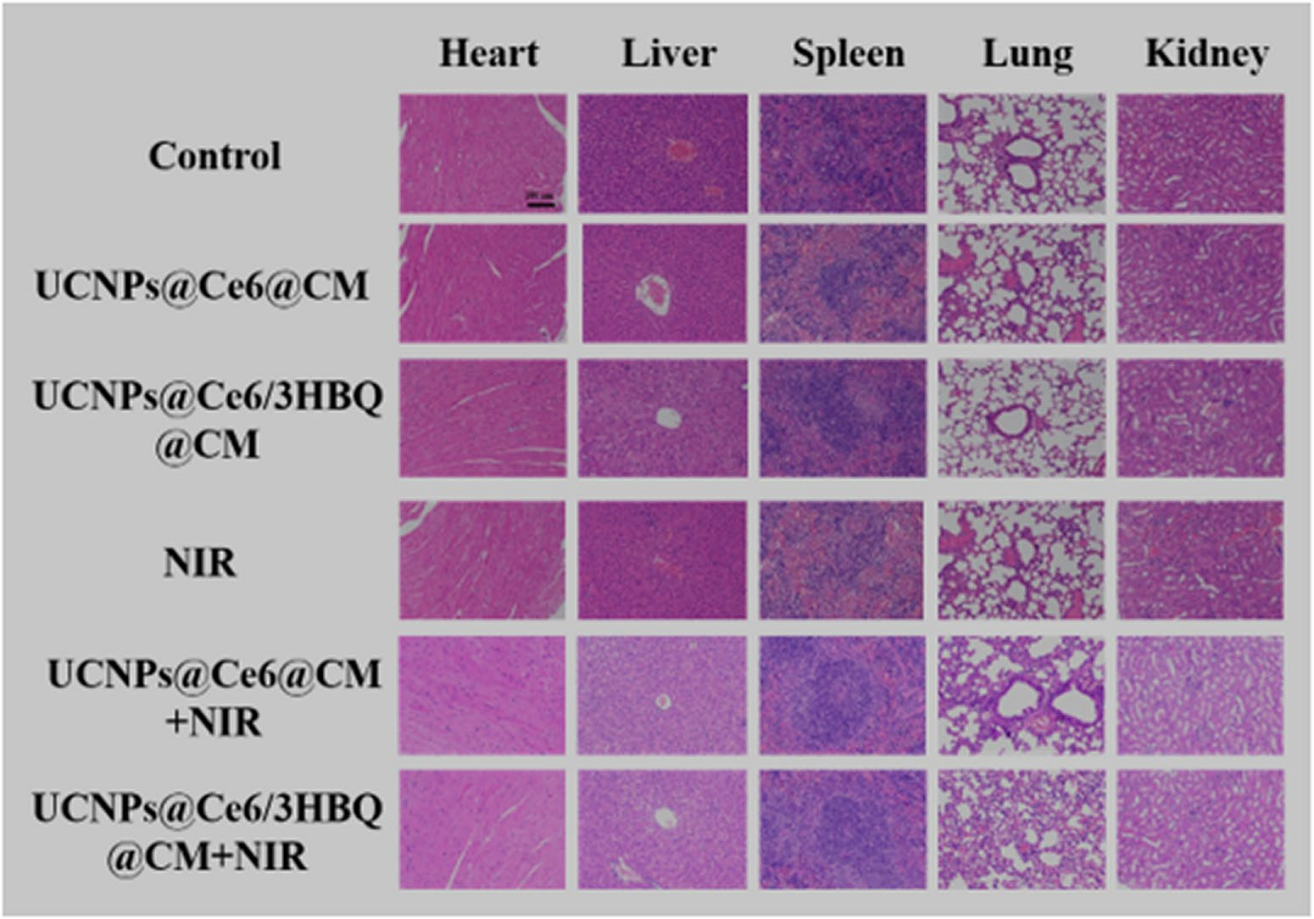


Corrected Fig. S36

**Figure:**
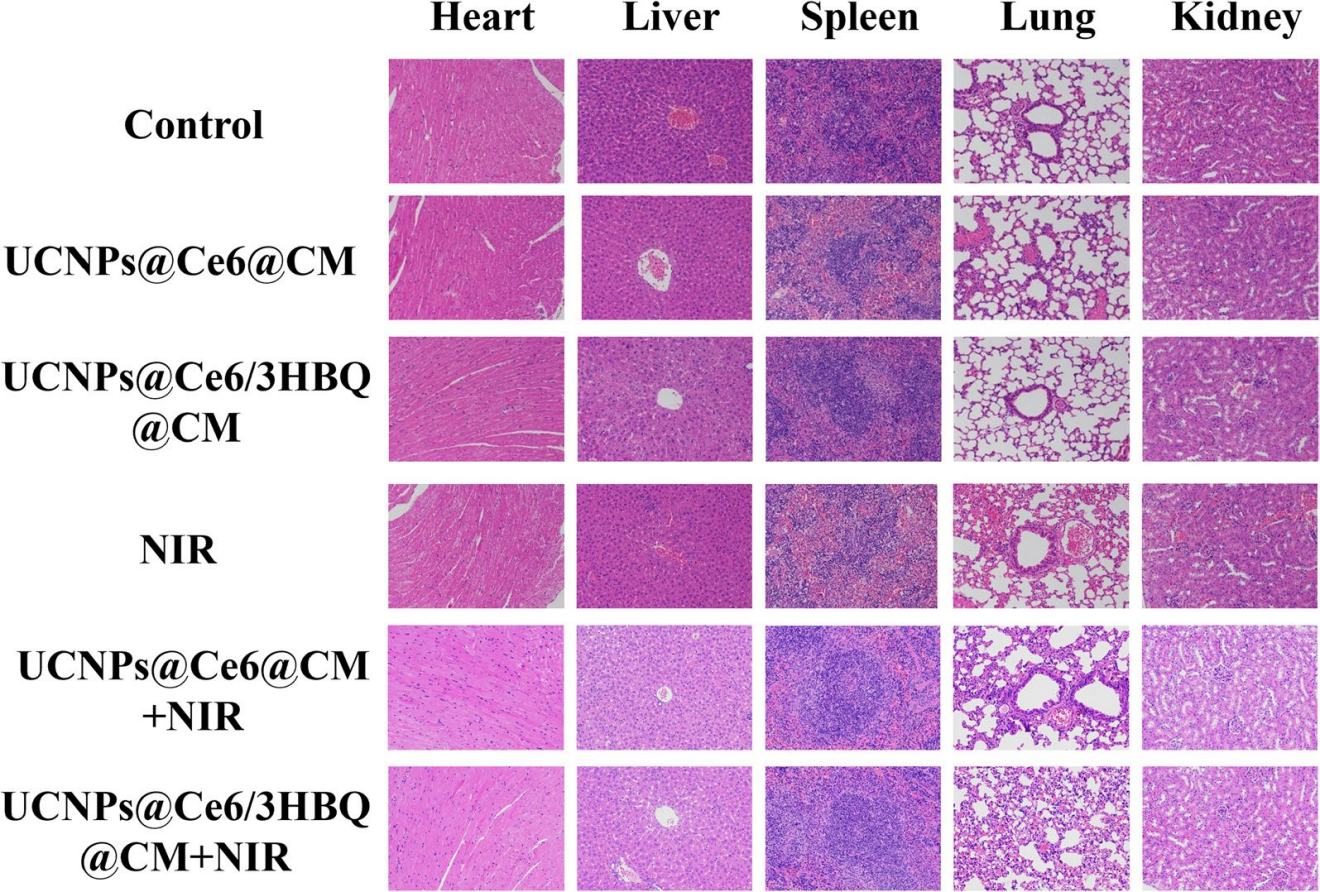
Figure S36. H&E stained major organs of U87MG-Luc glioma-bearing mice after receiving different treatments. Scale bar: 100 µm

