# Supplementary material for: Near-infrared light triggered in situ release of CO for enhanced therapy of glioblastoma
Source: J Nanobiotechnology. 2023 Feb 9;21:48. doi: 10.1186/s12951-023-01802-9 (PMC9912522; doi:10.1186/s12951-023-01802-9)
Supplement: Supplementary file 1 — Additional file 1: Figure S1. 1H-NMR spectrum of 3HBQ. Figure S2. 13C-NMR spectrum of 3HBQ. Figure S3. High resolution mass spectrum (HRMS) of 3HBQ. Figure S4. 3HBQ solution light response releases CO. Figure S5. The fluorescence emission spectra of 3HBQ solution after being irradiated with a 450-nm LED light source. Figure S6. The mechanism of CO detecting by FL-CO. Figure S7. The fluorescence spectra of FL-CO incubated with 3HBQ and irradiated for different times in 50% DMSO-PBS buffer. Figure S8. HRMS of the product obtained by irradiating 3HBQ. Figure S9. The mechanism of CO release by 3HBQ. Figure S10. The histograms of the size distribution of (a) C, (b) C-S1, (c) C-S1-S2, (d) C-S1-S2-S3, (e) C-S1-S2-S3-S4 structured UCNPs. Figure S11. HRTEM image of OA-UCNPs. Figure S12. Constituent elements and contents of OA-UCNPs. Figure S13. Upconversion emission spectrum of UCNPs under 980-nm laser excitation. Figure S14. FTIR spectra of OA-UCNPs, PC and PC-UCNPs. Figure S15. The TEM image (a) and histograms of the size distribution (b) of UCNPs@Ce6/3HBQ. Figure S16. UV–vis absorption spectra of (a) Ce6 and (c) 3HBQ with different concentrations in DMSO. Figure S17. UV–visible absorption spectra of UCNPs@Ce6/3HBQ and the supernatant after centrifugation within 48 h. Figure S18. Detection of reactive oxygen species production by UCNPs@Ce6 and CO release by UCNPs@3HBQ. Figure S19. Control experiments to exclude the direct effect of NIR on 3HBQ and Ce6 molecules. Figure S20. UCNPs@Ce6/3HBQ stability test. Figure S21. UCNPs@Ce6/3HBQ cell uptake imaging. Figure S22. Expression levels of HMOX-1, AKT-1 and NRF-2 in U87MG cells after receiving different treatments. Figure S23. ATP levels in U87MG cells after receiving different treatments. Figure S24. Expression levels of TNF-α and IL-6 in U87MG cells after receiving different treatments. Figure S25. Zeta potential of 1) CM, 2) UCNPs@Ce6/3HBQ and 3) UCNPs@Ce6/3HBQ@CM. Figure S26. Hydrodynamic diameter of UCNPs@Ce6/3HBQ and UCNPs [file 12951_2023_1802_MOESM1_ESM.docx]

**
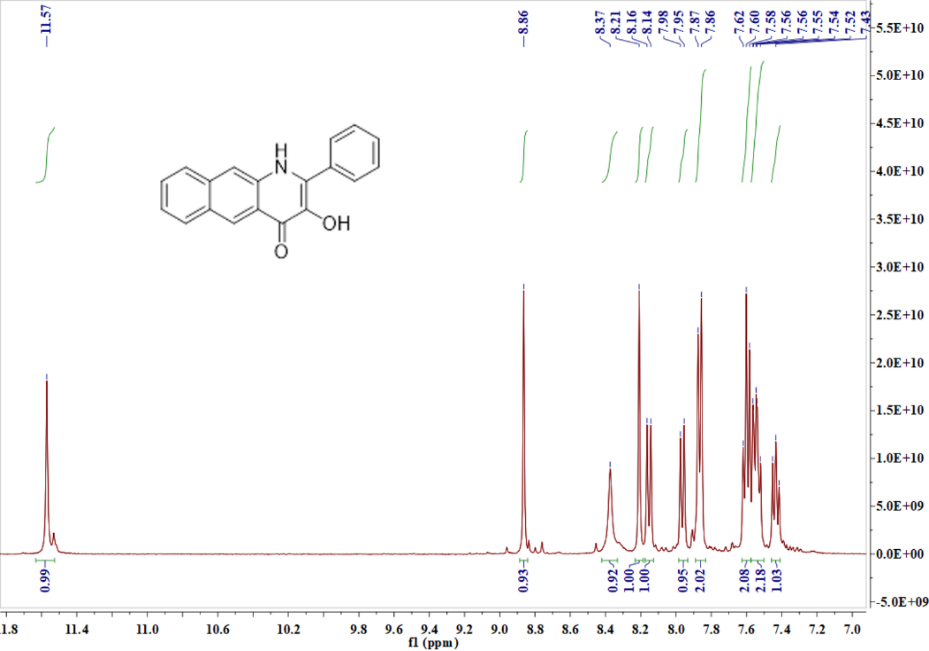
**

**Figure S1.** ^1^H-NMR spectrum of 3HBQ. ^1^H NMR (400 MHz, DMSO-d6) δ 11.57 (s, 1H), 8.86 (s, 1H), 8.37 (s, 1H), 8.21 (s, 1H), 8.15 (d, J = 8.5 Hz, 1H), 7.96 (d, J = 8.4 Hz, 1H), 7.87 (d, J = 7.1 Hz, 2H), 7.60 (t, J = 7.3 Hz, 2H), 7.57-7.50 (m, 2H), 7.46-7.41 (m, 1H).

**
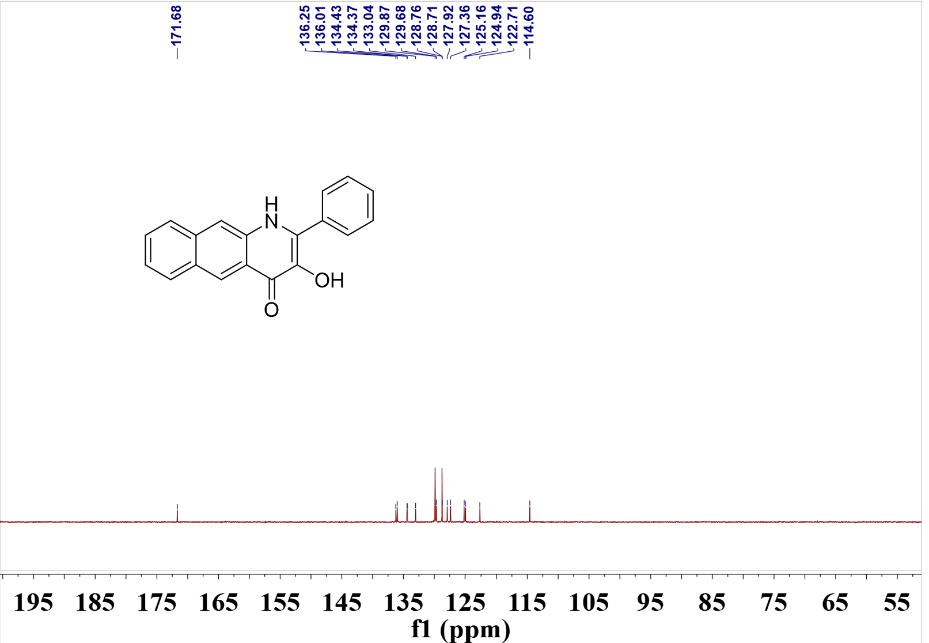
**

**Figure S2.** ^13^C-NMR spectrum of 3HBQ. ^13^C NMR (101 MHz, DMSO) δ 171.68, 136.25, 136.01, 134.43, 134.37, 133.04, 129.87, 129.68, 128.76, 128.71, 127.92, 127.36, 125.16, 124.94, 122.71, 114.60.


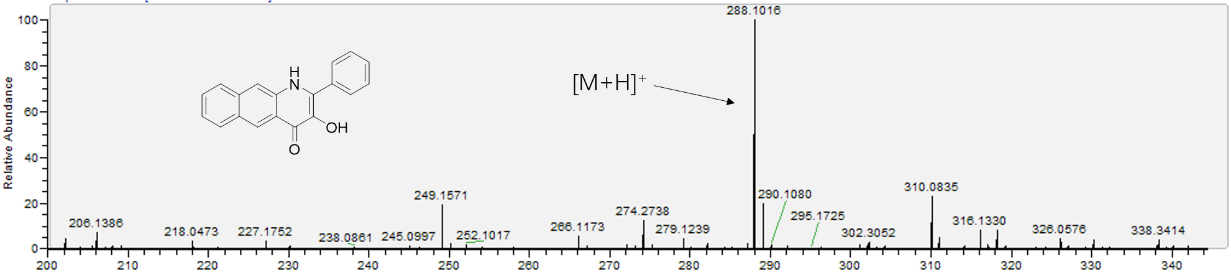


**Figure S3.** High resolution mass spectrum (HRMS) of 3HBQ. HRMS (m/z): [M+H]^+^ calcd for [C_19_H_14_NO_2_]^+^ 288.1019, found 288.1016.


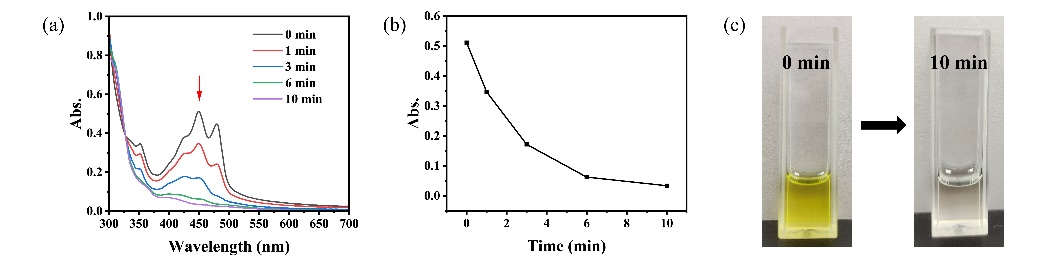


**Figure S4.** (a) UV-vis absorption spectra of 3HBQ solution (100 μM) upon irradiation with a 450-nm LED light for different times. 3HBQ was dissolved in a 5% DMSO-PBS buffer (10 mM, pH=7.4) in the presence of 20 mM CTAB. (b) Dependence of the absorbance at 450 nm of 3HBQ on the irradiation time. (c) Color change of 3HBQ solution after irradiation with LED light source.


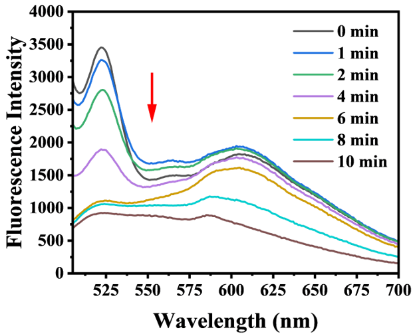


**Figure S5.** The fluorescence emission spectra of 3HBQ solution (100 μM) after being irradiated with a 450-nm LED light source. 3HBQ was dissolved in 5% DMSO-PBS buffer (10 mM, pH = 7.4) in the presence of 20 mM CTAB (λ_ex_ = 428 nm).


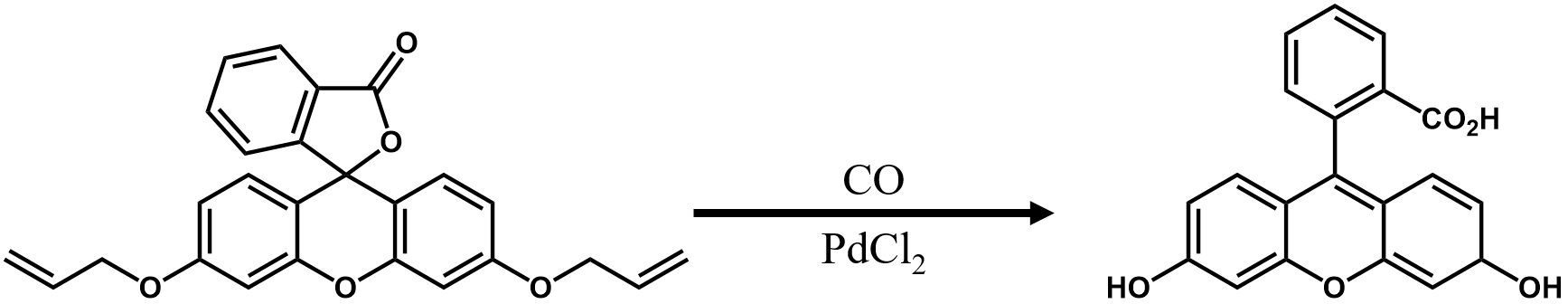


**Figure S6.** The mechanism of CO detecting by FL-CO.


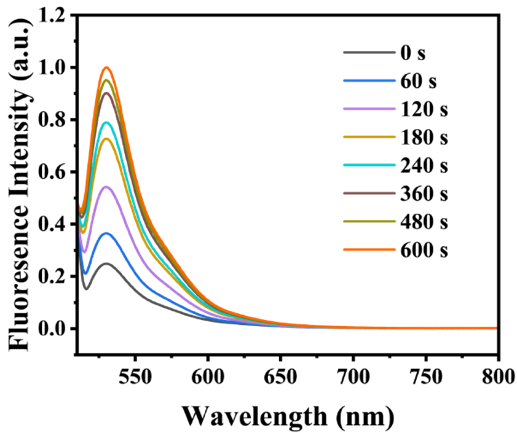


**Figure S7.** The fluorescence spectra of FL-CO incubated with 3HBQ and irradiated for different times in 50% DMSO-PBS buffer (10 mM, pH = 7.4, λ_ex_ = 500 nm).


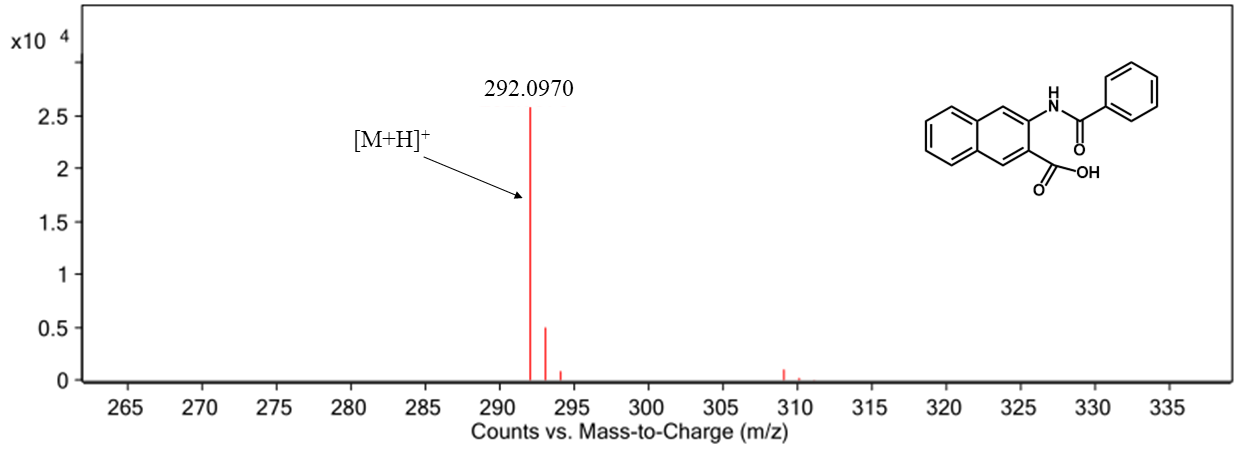


**Figure S8.** HRMS of the product obtained by irradiating 3HBQ. HRMS (m/z): [M+H]^+^ calcd for [C_18_H_14_NO_3_]^+^ 292.0968, found 292.0970.


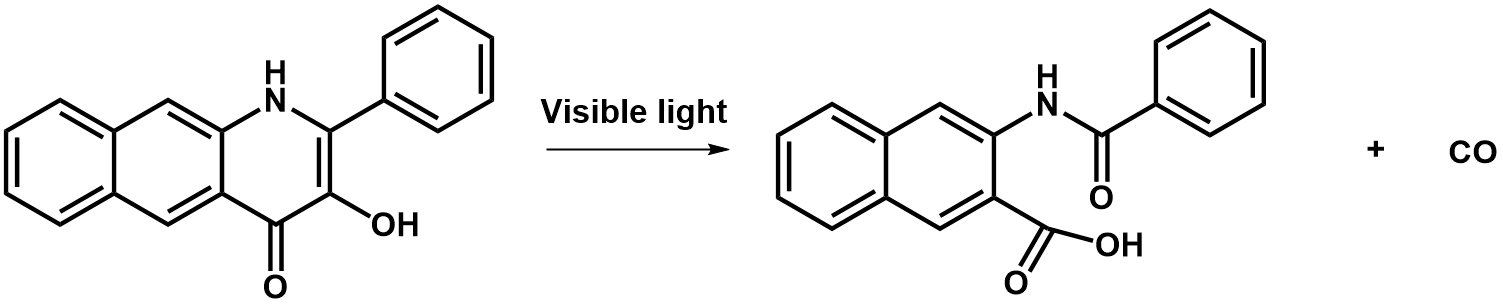


**Figure S9.** The mechanism of CO release by 3HBQ.


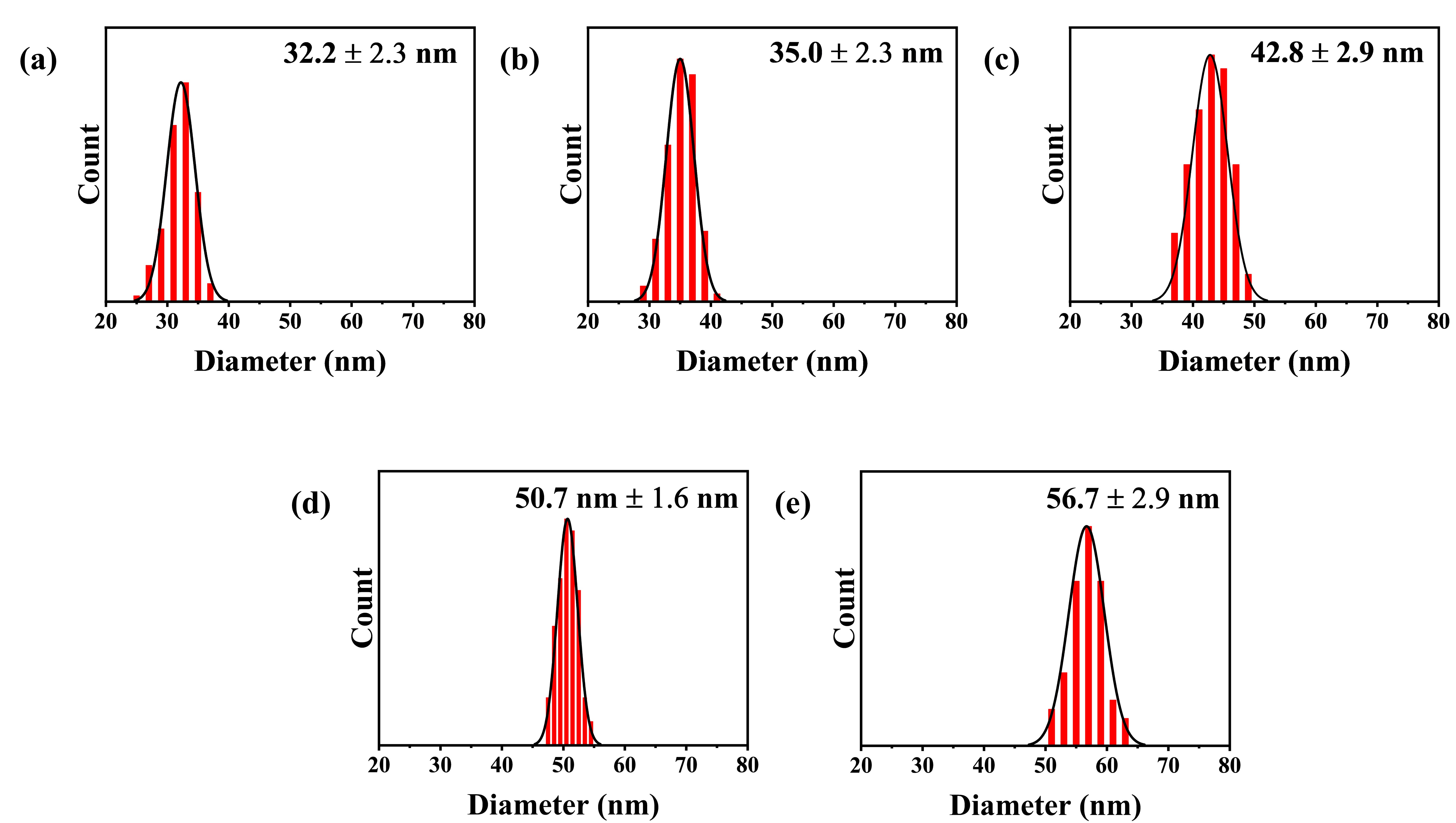


**Figure S10.** The histograms of the size distribution of (a) C, (b) C-S1, (c) C-S1-S2, (d) C-S1-S2-S3, (e) C-S1-S2-S3-S4 structured UCNPs.


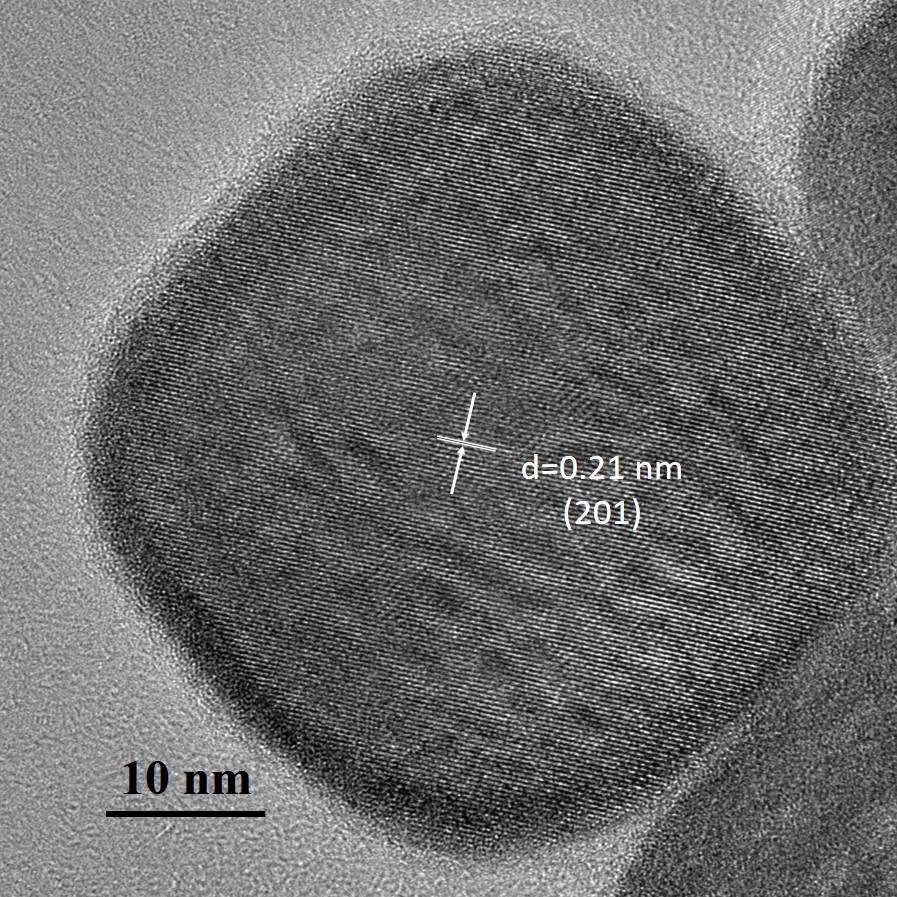


**Figure S11.** HRTEM image of OA-UCNPs.


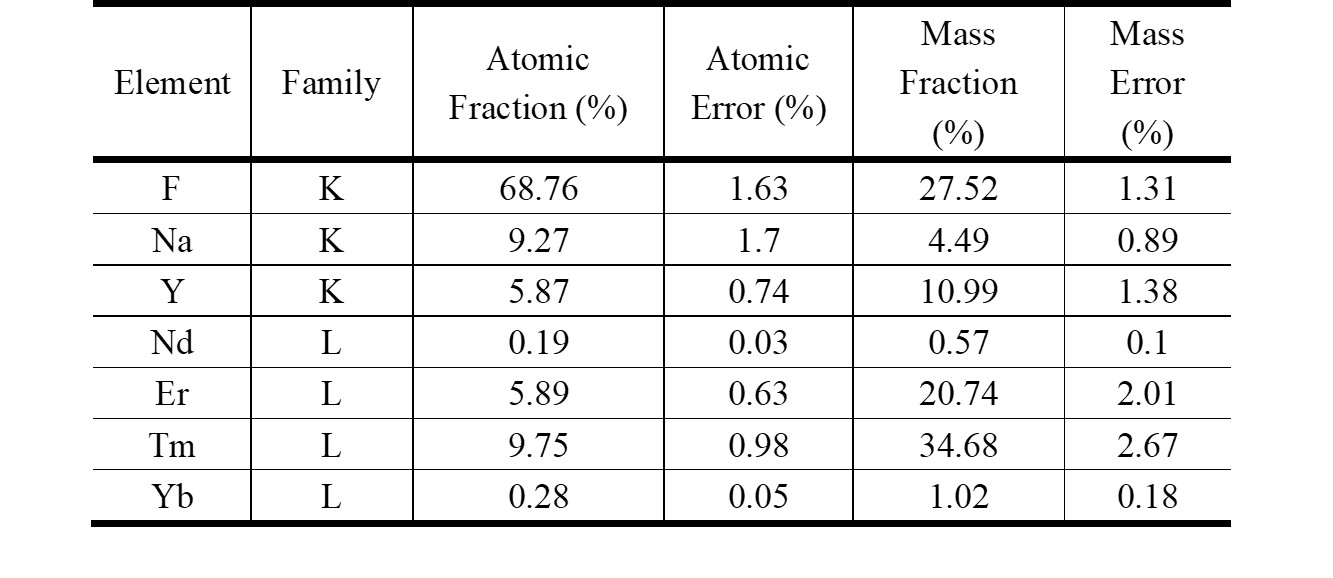


**Figure S12.** Constituent elements and contents of OA-UCNPs.

**
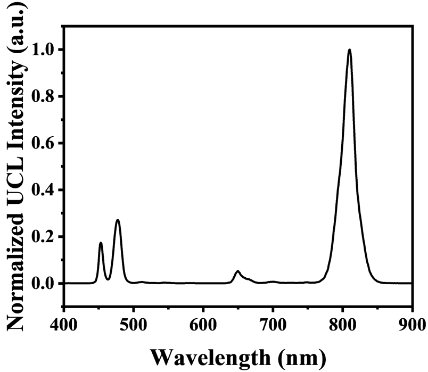
**

**Figure S13.** Upconversion emission spectrum of UCNPs under 980-nm laser excitation.

**
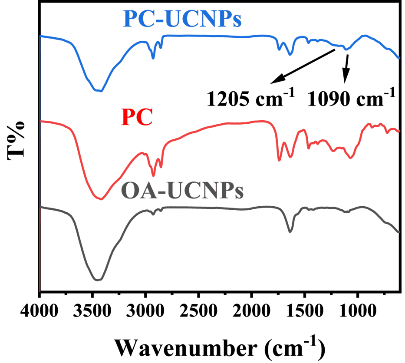
**

**Figure S14.** FTIR spectra of OA-UCNPs, PC and PC-UCNPs.


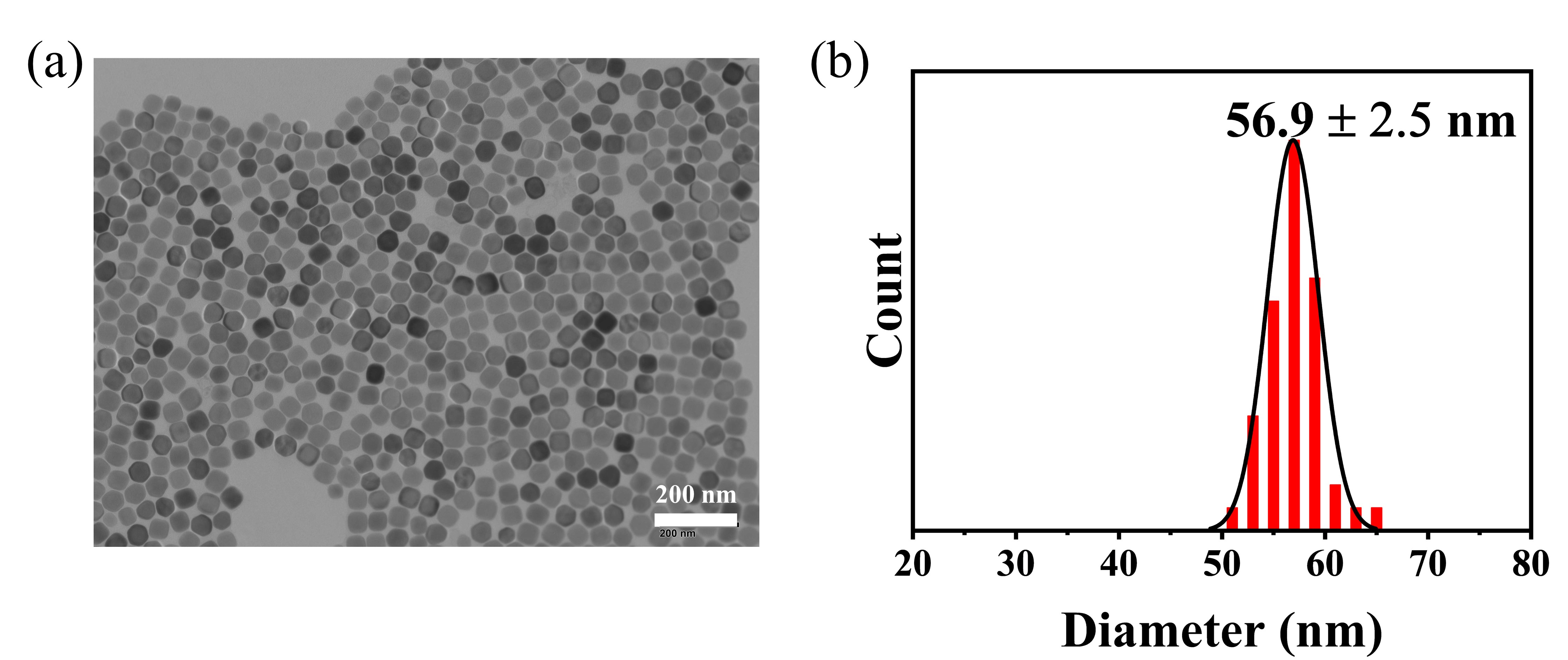


**Figure S15.** The TEM image (a) and histograms of the size distribution (b) of UCNPs@Ce6/3HBQ.


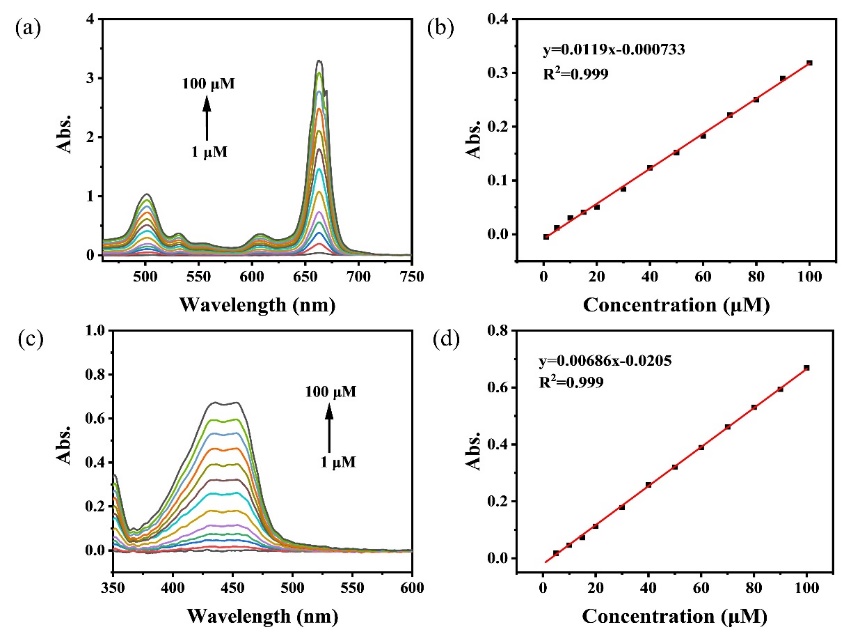


**Figure S16.** UV-vis absorption spectra of (a) Ce6 and (c) 3HBQ with different concentrations in DMSO. The plot of absorbance at (b) 650 nm and (d) 450 nm against the concentration of Ce6 and 3HBQ, respectively.


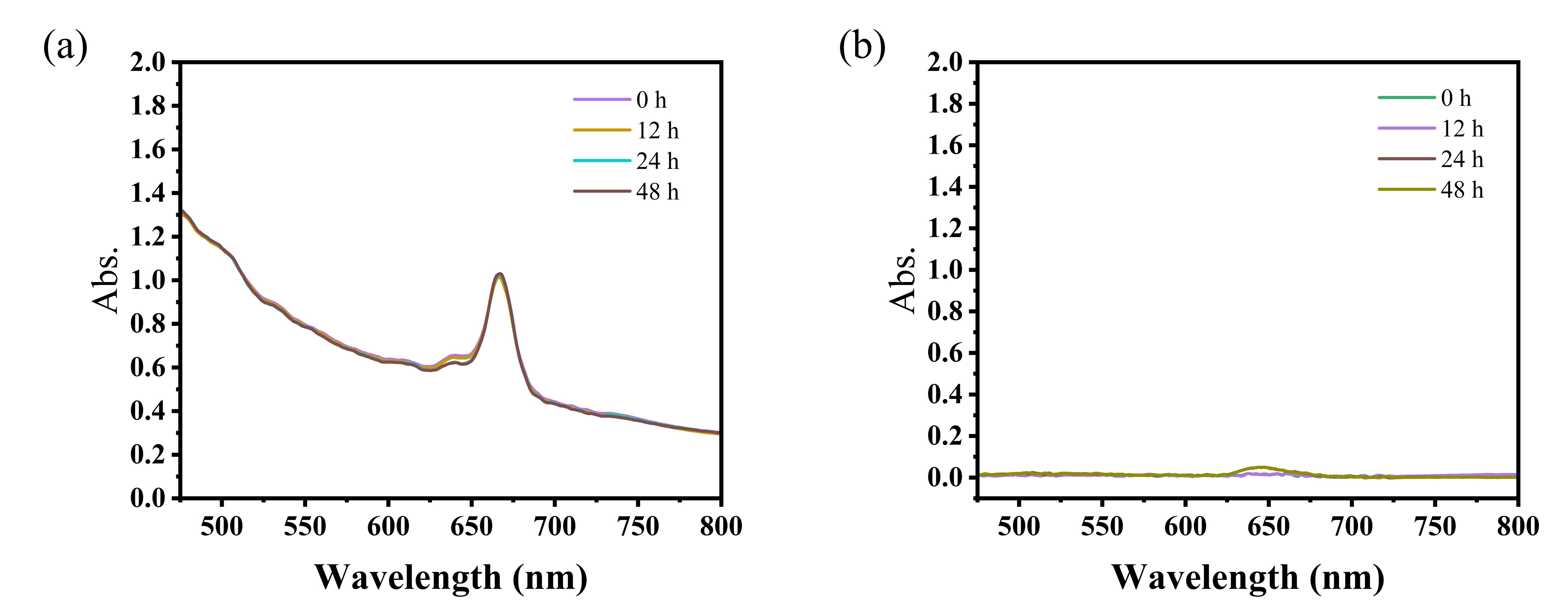


**Figure S17**. UV-visible absorption spectra of UCNPs@Ce6/3HBQ (a) and the supernatant after centrifugation (b) within 48 hours.


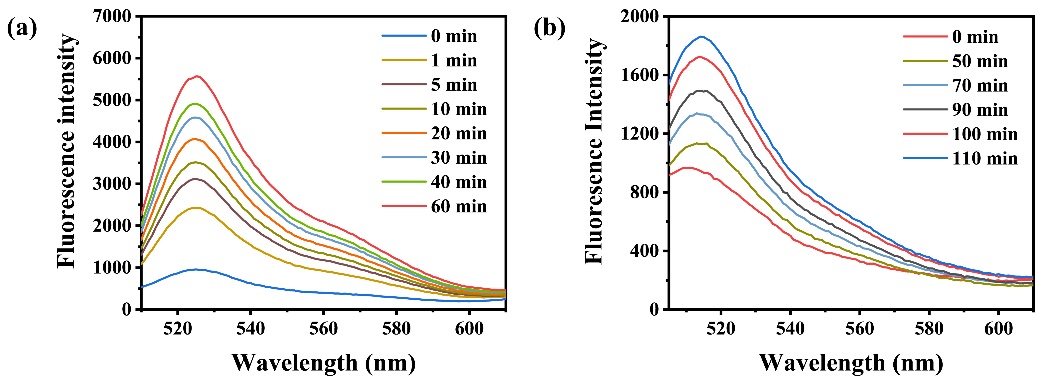


**Figure S18.** (a) Fluorescence change of DCFH after incubated with UCNPs@Ce6 and irradiated with an 808-nm laser (0.3 W/cm^2^). (b) Fluorescence change of CO probe system (5 μM FL-CO + 5 μM PdCl_2_) after incubated with UCNPs@3HBQ and irradiated with an 808-nm laser (0.3 W/cm^2^).


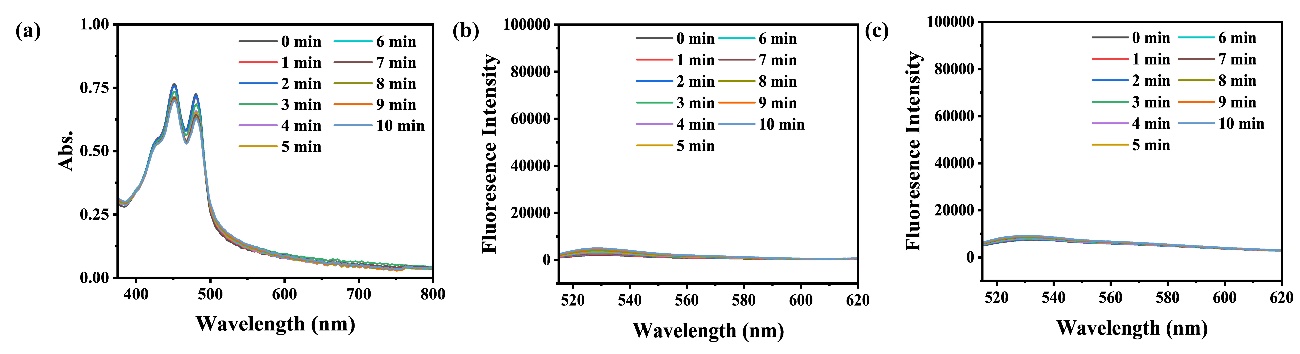


**Figure S19.** (a) UV-vis absorption spectra of 3HBQ solution (100 μM) and (b) emission spectra of DCFH (10 μM) with 808-nm laser irradiation (0.3 W/cm^2^) at different time points. (c) Emission spectra of DCFH (10 μM) incubated with PC-UCNPs with 808-nm laser irradiation (0.3 W/cm^2^) at different time points. 3HBQ was dissolved in a 5% DMSO-PBS buffer (10 mM, pH=7.4) in the presence of 20 mM CTAB. Ce6 was dissolved in HEPES (10 mM, pH=7.4) buffer solution.


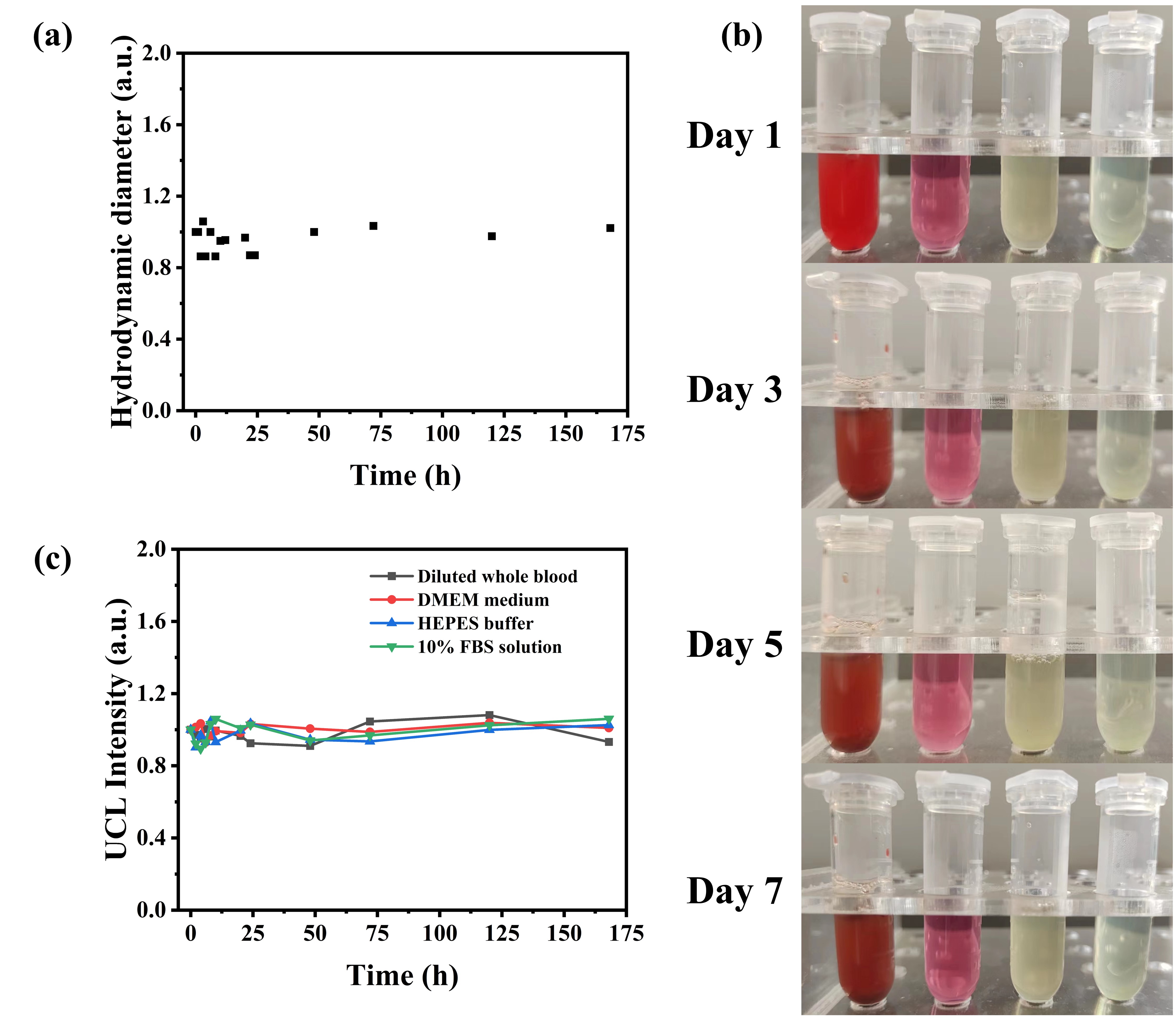


**Figure S20.** (a) Hydrodynamic diameter of UCNPs@Ce6/3HBQ in HEPES buffer at different time points. (b) Optical images of UCNPs@Ce6/3HBQ in different media including 1) 20-fold diluted whole blood, 2) DMEM, 3) 10% fetal bovine serum, and 4) HEPES buffer (pH=7.4, 10 mM). (c) The UCL intensities of UCNPs@Ce6/3HBQ at 450 nm under 808-nm laser excitation after incubation at 37°C in different media for different time.


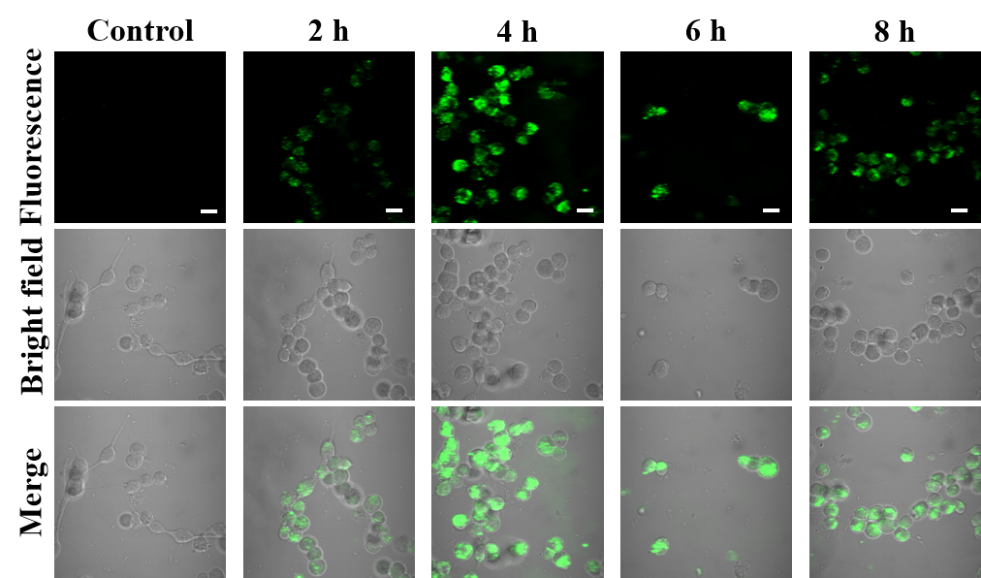


**Figure S21.** CLSM images of U87MG cells incubated with UCNPs@Ce6/3HBQ for 2, 4, 6 and 8 h. Images were collected at 400-500 nm with an 808-nm laser excitation. Scale bar: 20 µm.


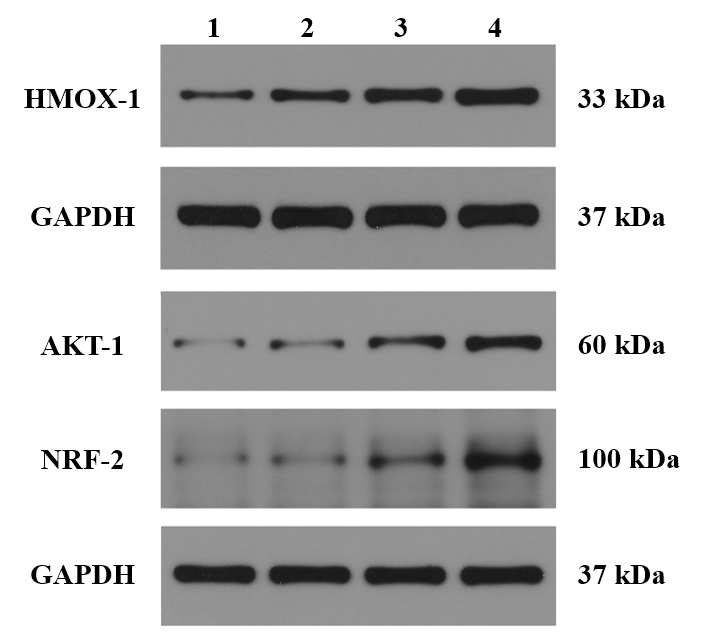


**Figure S22.** Expression levels of HMOX-1, AKT-1 and NRF-2 in U87MG cells after receiving different treatments: 1) Control, 2) UCNPs@Ce6+NIR, 3) UCNPs@3HBQ+NIR, 4) UCNPs@Ce6/3HBQ+NIR.


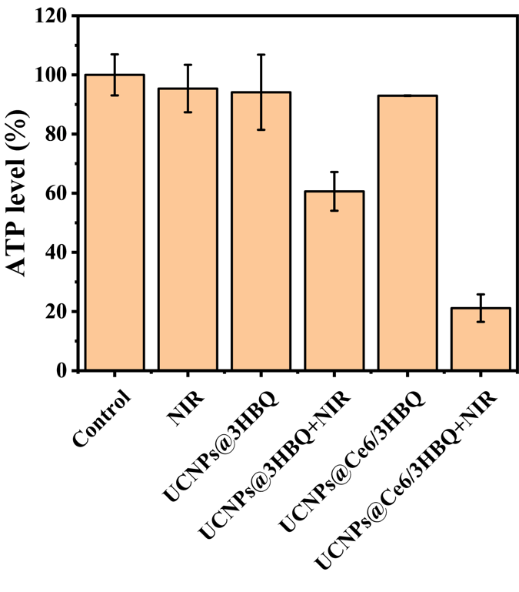


**Figure S23.** ATP levels in U87MG cells after receiving different treatments.


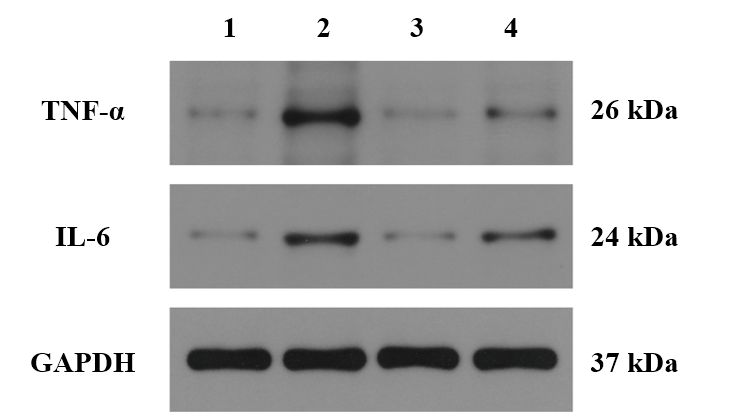


**Figure S24.** Expression levels of TNF-α and IL-6 in U87MG cells after receiving different treatments: 1) Control, 2) UCNPs@Ce6+NIR, 3) UCNPs@3HBQ+NIR, 4) UCNPs@Ce6/3HBQ+NIR.


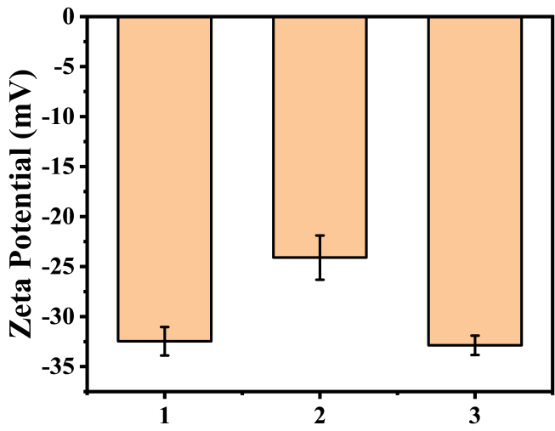


**Figure S25.** Zeta potential of 1) CM, 2) UCNPs@Ce6/3HBQ and 3) UCNPs@Ce6/3HBQ@CM.


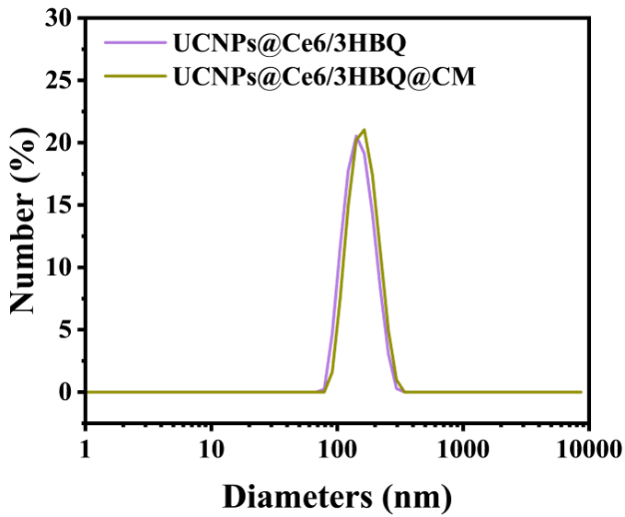


**Figure S26.** Hydrodynamic diameter of UCNPs@Ce6/3HBQ and UCNPs@Ce6/3HBQ@CM.


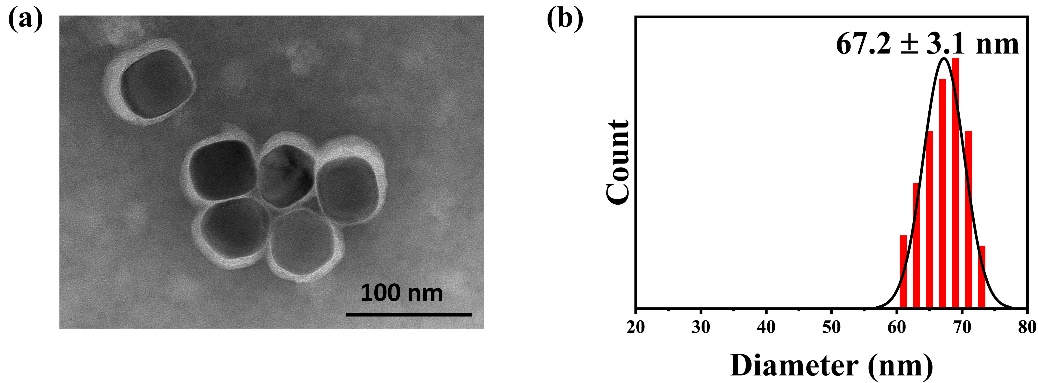


**Figure S27.** The TEM image and histogram of the size distribution of UCNPs@Ce6/3HBQ@CM.


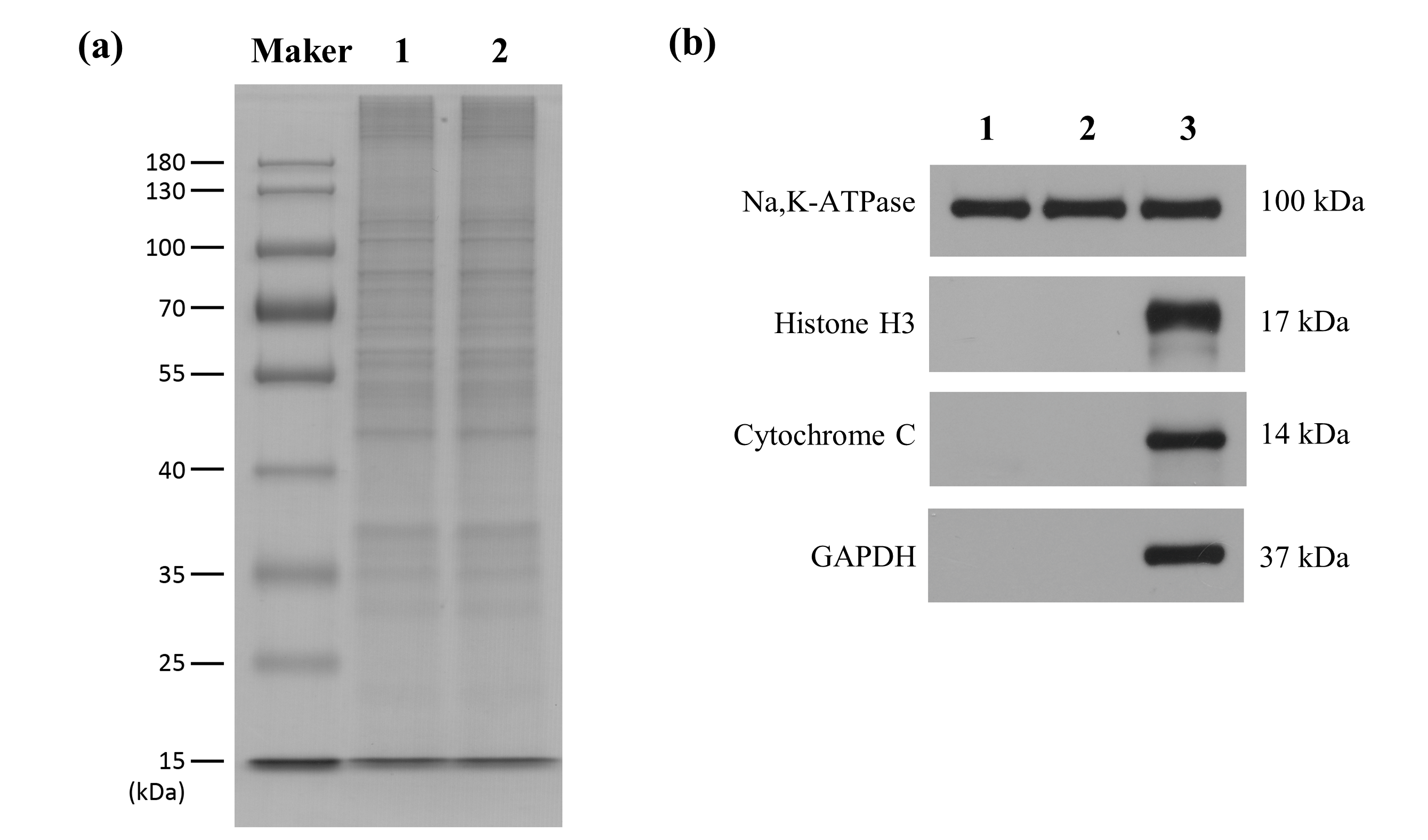


**Figure S28.** (a) SDS-PAGE protein analysis of U87MG cells membrane (1), UCNPs@Ce6/3HBQ@CM) (2). (b) Western blot analysis of the expression of Na^+^/K^+^ ATPase (cell membrane marker), histone H3 (nuclear marker), Cytochrome C (mitochondrial marker), and GAPDH (cytosolic marker) in (1) U87MG cells membrane, (2) UCNPs@Ce6/3HBQ@CM and (3) U87MG cells..


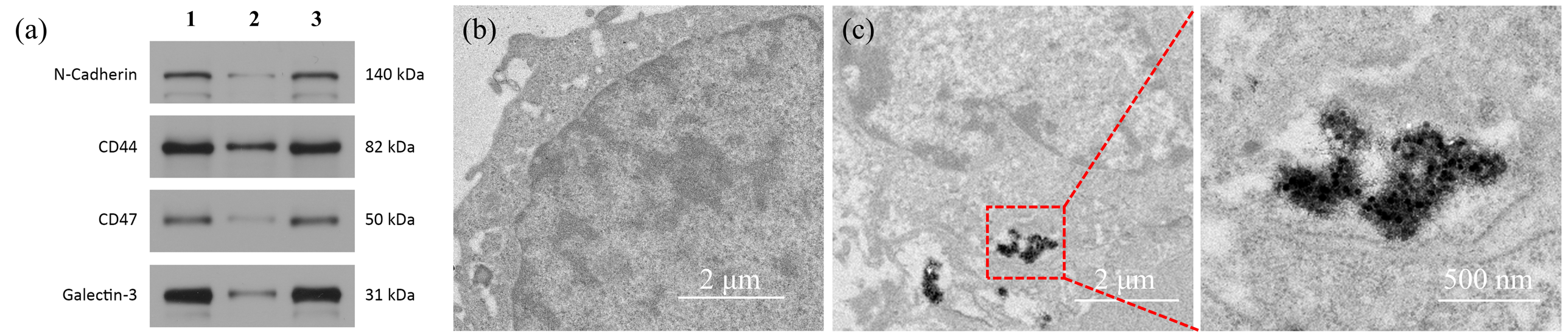


**Figure S29.** (a) Western blot analysis of the expression of membrane proteins (N-cadherin, CD44, CD47, Galectin-3) in (1) U87MG cells membrane, (2) UCNPs@Ce6/3HBQ@CM; (3) U87MG cells. TEM images of U87MG cell incubated with (b) UCNPs@Ce6/3HBQ and (c) UCNPs@Ce6/3HBQ@CM.


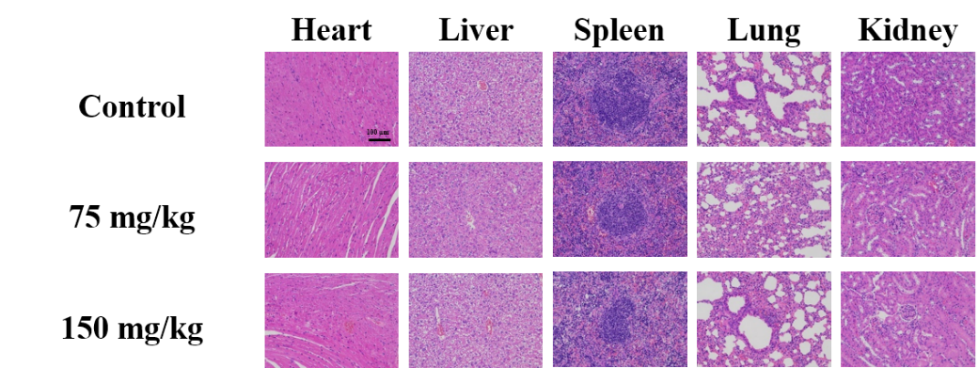


**Figure S30.** H&E staining of major organs (healthy mice) after injection with different dosages of UCNPs@Ce6/3HBQ@CM. Scale bar: 100 µm.


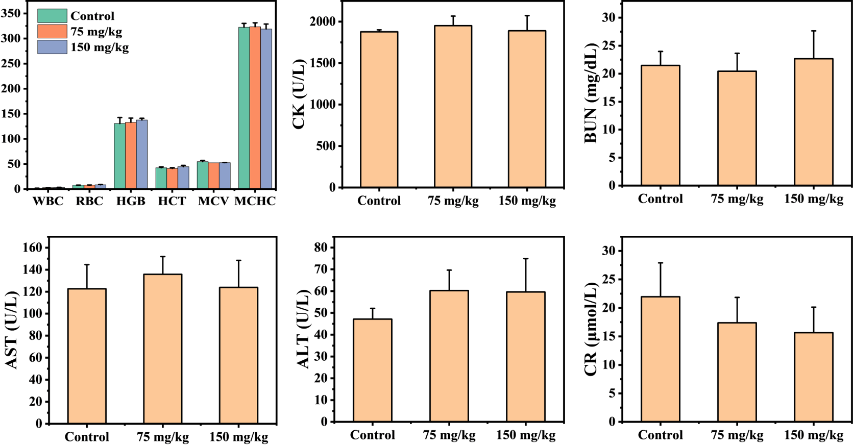


**Figure S31.** Blood routine and serum biochemical levels of mice after injection with different doses of UCNPs@Ce6/3HBQ@CM (0, 75 and 150 mg/kg body weight) including white blood cell count (WBC), red blood cell count (RBC) hemoglobin (HGB), hematocrit (HCT), mean vascular volume (MCV), mean vascular hemoglobin concentration (MCHC), creatine kinase (CK), blood urea nitrogen (BUN), aspartate aminotransferase (AST), alanine aminotransferase (ALT), and creatinine (CR).


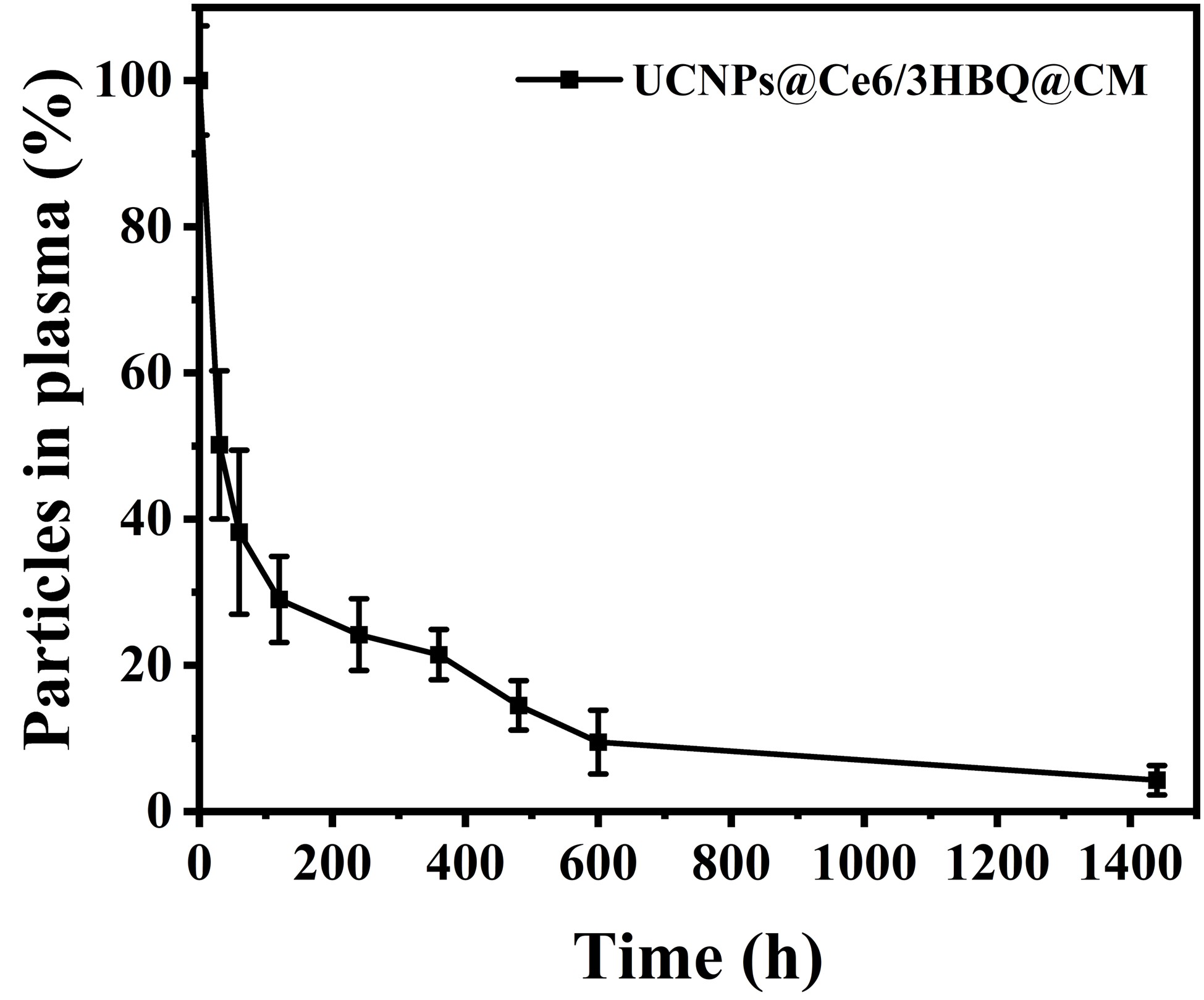


**Figure S32.** Blood concentration-time curve of UCNPs@Ce6/3HBQ@CM in mice.


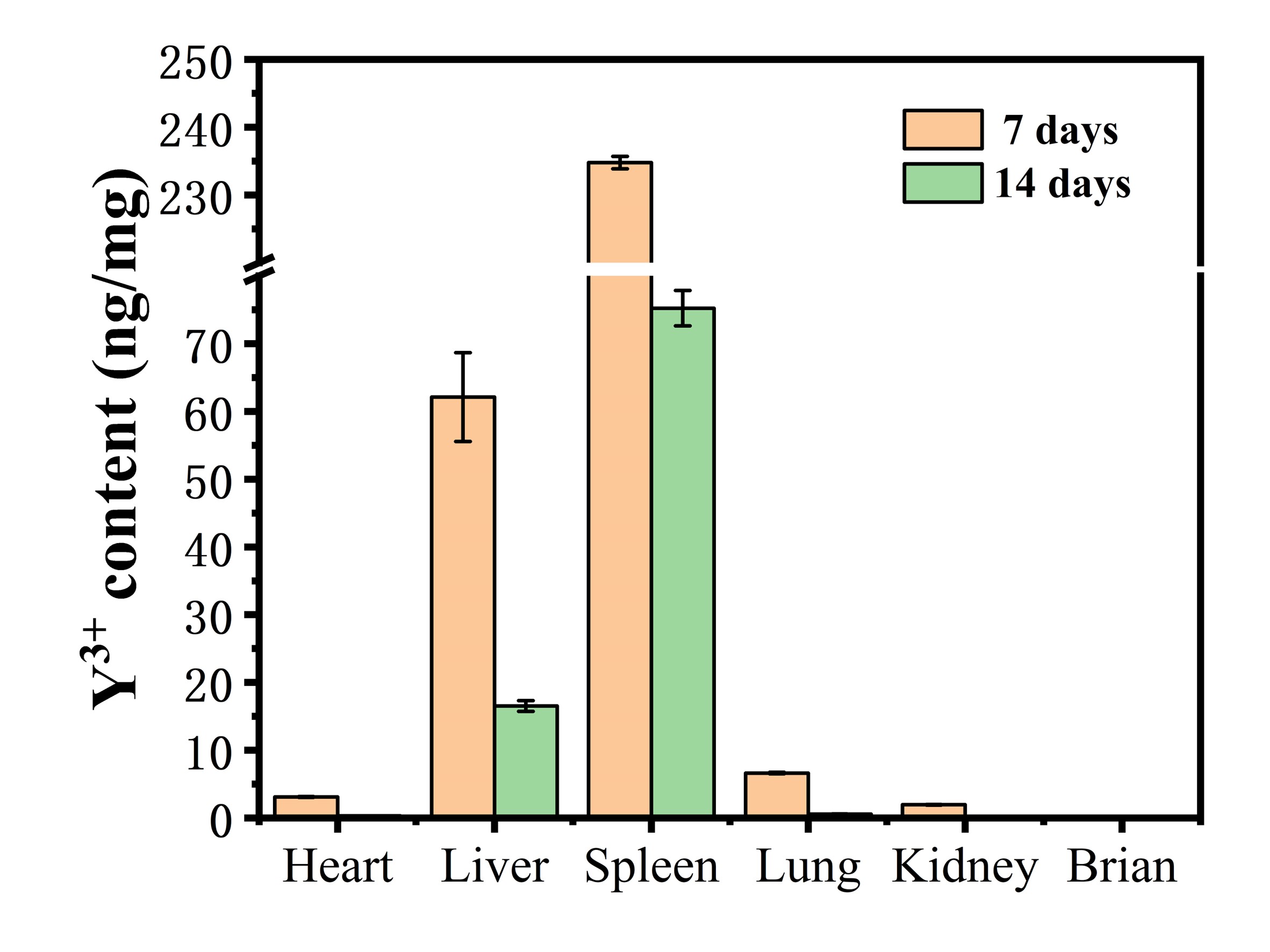


**Figure S33.** Y^3+^ content in major organs of mice after injection of UCNPs@Ce6/3HBQ@CM for 7 days and 14 days.


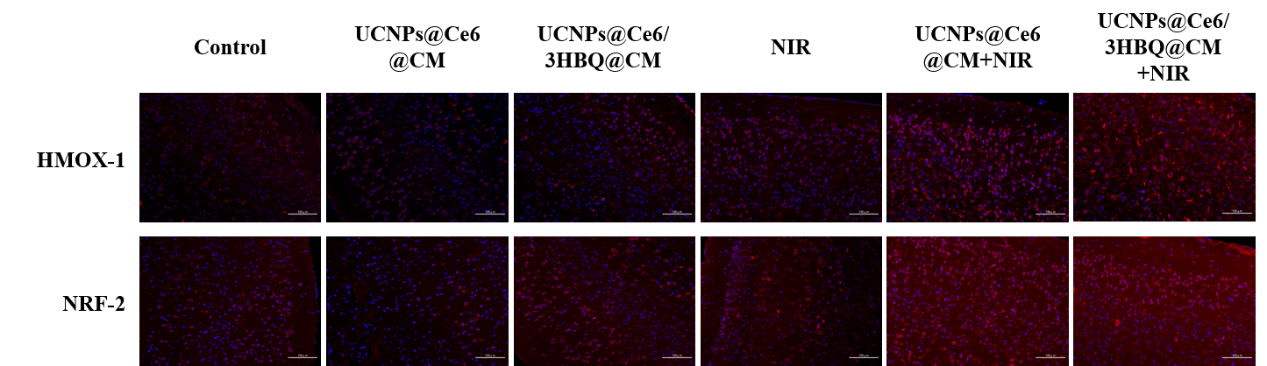


**Figure S34.** HMOX-1 and NRF-2 levels of GBM site on the 15^th^ day. Scale bar: 100 µm.


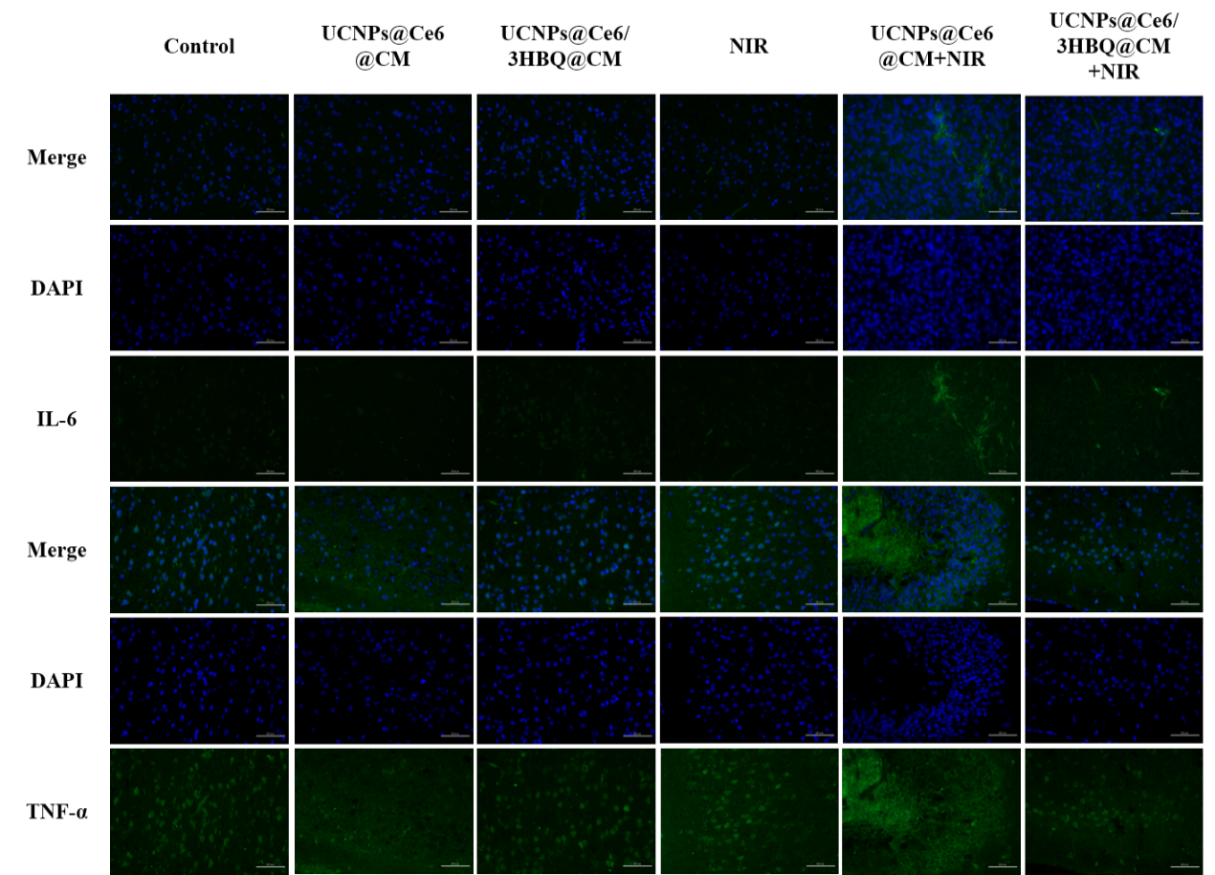


**Figure S35.** TNF-α and IL-6 levels of GBM site on the 15^th^ day. Scale bar: 50 µm.


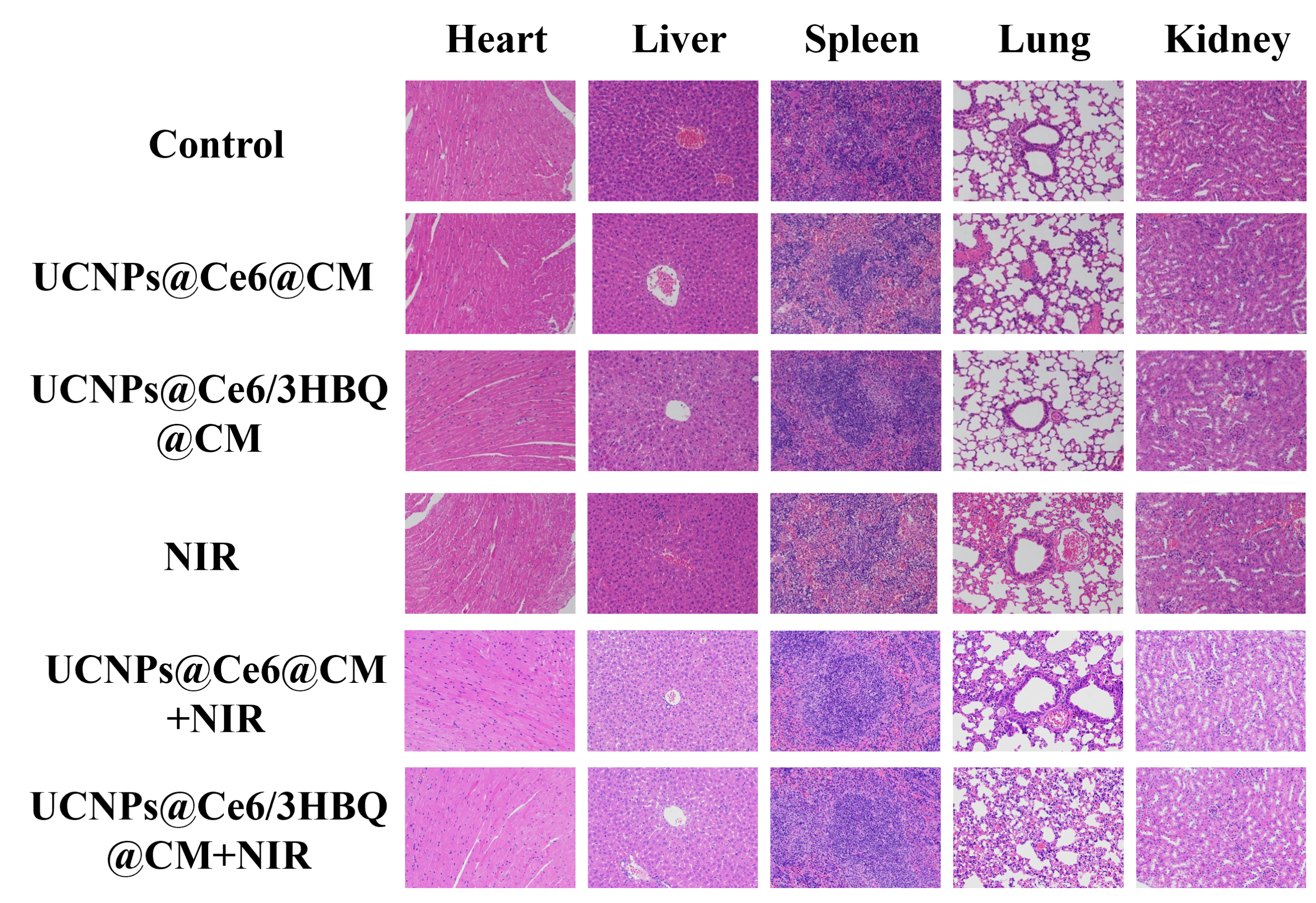


**Figure S36.** H&E stained major organs of U87MG-Luc glioma-bearing mice after receiving different treatments. Scale bar: 100 µm.
